# Glaulactams A–C, daphniphyllum alkaloids from *Daphniphyllum glaucescens*

**DOI:** 10.1038/s41598-018-33748-6

**Published:** 2018-10-18

**Authors:** Chih-Hua Chao, Ju-Chien Cheng, Théo P. Gonçalves, Kuo-Wei Huang, Chi-Chien Lin, Hui-Chi Huang, Syh-Yuan Hwang, Yang-Chang Wu

**Affiliations:** 1 School of Pharmacy, China Medical University, Taichung, 404 Taiwan; 2 Chinese Medicine Research and Development Center and Department of Medical Research, China Medical University Hospital, Taichung, 404 Taiwan; 3 Department of Medical Laboratory Science and Biotechnology, China Medical University, Taichung, 404 Taiwan; 4 Division of Physical Sciences and Engineering and KAUST Catalysis Center, King Abdullah University of Science and Technology, Thuwal, 23955-6900 Saudi Arabia; 5 Institute of Biomedical Science, National Chung-Hsing University, Taichung, 402 Taiwan; 6 Department of Health and Nutrition, Asia University, Taichung, 413 Taiwan; 7 Department of Chinese Pharmaceutical Sciences and Chinese Medicine Resources, China Medical University, Taichung, 404 Taiwan; 8 Endemic Species Research Institute, Council of Agriculture, Nantou, 552 Taiwan; 9 Graduate Institute of Natural Products and Research Center for Natural Products & Drug Development, Kaohsiung Medical University, Kaohsiung, 807 Taiwan; 10 Department of Medical Research, Kaohsiung Medical University Hospital, Kaohsiung, 807 Taiwan

## Abstract

Glaulactams A–C (1–3), which possess a novel skeleton, as well as the known compound daphmanidin B (4), were isolated from the leaves of *Daphniphyllum glaucescens* and separated using ion-exchange chromatography aided by NMR fingerprinting. Their structures, including their absolute configurations, were elucidated by spectroscopic analyses and time-dependent density-functional-theory-calculated electronic circular dichroism spectra; the data were subsequently analyzed to gain insight into the respective biogenetic relationships between the isolates, which exhibited anti-H1N1 and immunosuppressive activities.

## Introduction

The first daphniphylline-type alkaloid, daphniphylline, which possesses a polycyclic, C_30_-aliphatic structure, was discovered in 1966^1^. A number of novel structures from the Daphniphyllaceae family of plants were recently reported, including himalensine A, with its 13,14,22-trinorcalyciphylline A backbone^2^, himalensine B with its 22-nor-1,13-secodaphnicyclidin framework^2^, and macropodumines A–C with fused pentacyclic ring systems^3^. The complex and fascinating structures of these alkaloids render them synthetically challenging^4^. Many types of alkaloid are reported to be produced by *Daphniphyllum glaucescens*, including daphniglaucin C, which has a novel structure that contains hexahydroazulene and octahydroindole ring systems^5^ and daphniglaucins A and B, which have unique 1-azoniatetracyclo[5.2.2.0^1,6^0.^4,9^]undecane motifs^6^. In addition, daphniglaucins D–H possess fused hexacyclic skeletons, and daphniglaucins J and K are yuzurimine-type alkaloids that have previously been reported from this species^7^.

Natural product chemists are very interested in the discovery of novel structures with unique properties^8^. To date, a variety of methods have been used to facilitate the discovery of novel natural products, with examples including NMR-fingerprinting^8^ and LC-MS-guided methods^9^. Daphnezomine-F-type^10,11^ and daphmanidin-C-type^12^ alkaloids are rare groups of daphniphyllum alkaloids; they each possess a lactam functional group that is biogenetically derived through the oxidative cleavage of a yuzurimine C–C bond. The presence of lactams in these daphniphyllum compounds inspired us to develop a new method that combines ion-exchange chromatography (IXC) and NMR fingerprinting to screen for new alkaloids. Hence, in this article, we report the isolation, structural characterization, and biological evaluation of glaulactams A–C (1–3) from the leaves of *Daphniphyllum glaucescens*.

## Results and Discussion

**Figure 1:**
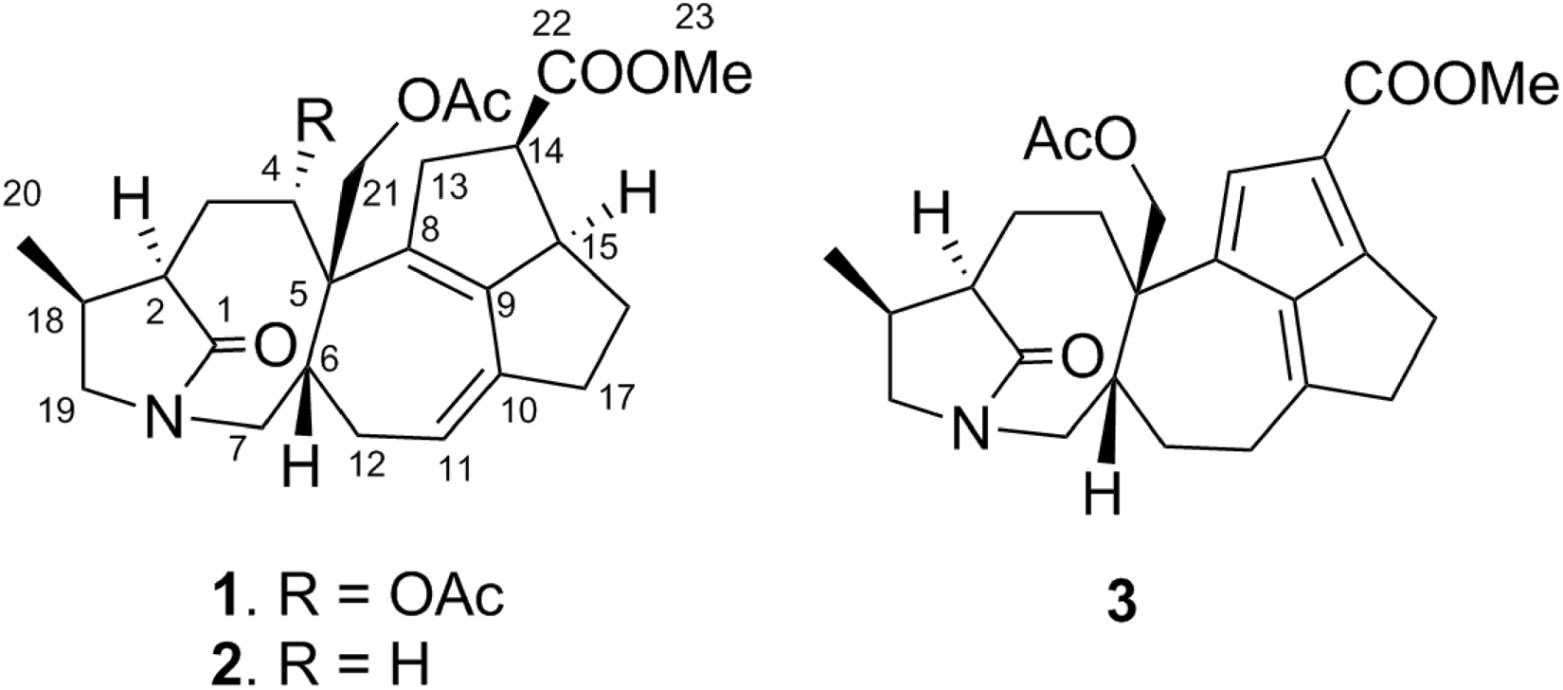
Structures of compounds 1–3.

Air-dried *D*. *glaucescens* leaves (1.53 kg) were extracted with MeOH and concentrated to obtain a crude extract, which was dispersed in 80% aqueous MeOH to give a methanolic suspension. This suspension was then partitioned with hexane (4 × 2 L) to remove the lipid constituents. The methanolic extract was filtered and passed through a cation-exchange resin. The eluent containing the acidic and neutral compounds was concentrated under reduced pressure and subjected to Diaion HP20 column chromatography, followed by vacuum liquid chromatography (VLC) over silica gel to yield 26 fractions (4A–4Z); among these fractions, the ^1^H NMR spectra of fractions 4V, 4W, and 4Z were found to exhibit signals characteristic of daphniphyllum alkaloids (see the Extraction and Isolation section). As a result, these three fractions were selected for repeated column chromatography over silica gel and RP-18 stationary phases to yield three compounds that had structures that were consistent with 1 (4.2 mg), 2 (29.2 mg), and 3 (3.0 mg) (Fig. 1), as described below.

**Figure 2:**
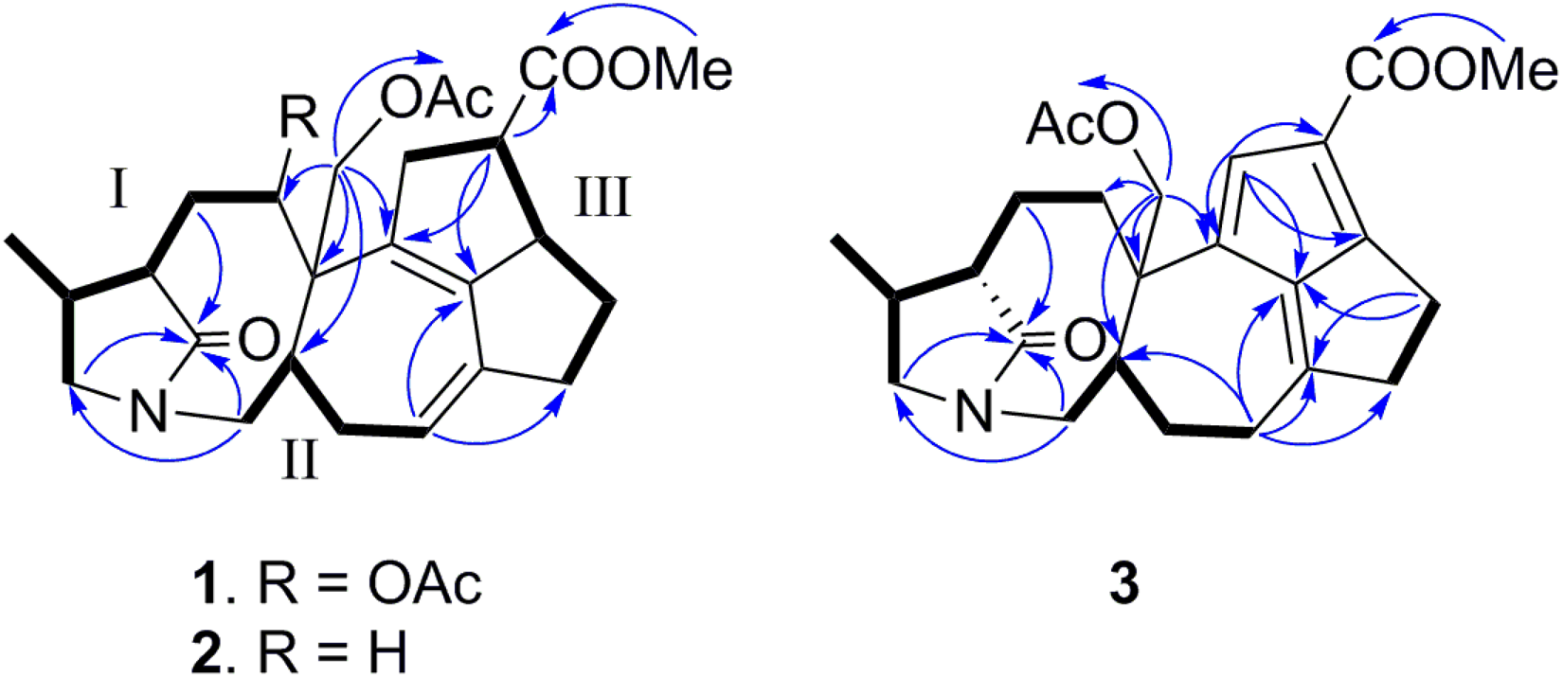
Selected ^1^H–^1^H COSY and HMBC correlations in 1–3.

Glaulactam A (1) exhibited a molecular ion peak at *m/z* 486.2494 [M + H]^+^ (calcd., 486.2486), consistent with the C_27_H_35_NO_7_ molecular formula and 11 degrees of unsaturation. The IR spectrum of 1 exhibited bands corresponding to carbonyl functionalities (1736, 1730, 1712, and 1695 cm^−1^). The ^13^C and ^1^H NMR data (Table 1) reveal the presence of 27 carbon signals ascribed to two acetyl groups (*δ*_C_ 168.9, 20.1, *δ*_H_ 2.05; *δ*_C_ 170.5, 20.4, *δ*_H_ 2.07), a methyl carboxylate (*δ*_C_ 173.9, 50.4, *δ*_H_ 3.59), a conjugated diene (*δ*_C_ 120.9, 134.1, 134.6, 143.3, *δ*_H_ 5.35), an acetoxy-bearing methine (*δ*_C_ 76.6, *δ*_H_ 5.51), an acetoxy-bearing methylene (*δ*_C_ 66.4, *δ*_H_ 4.06, 5.03), and an amide carbonyl (*δ*_C_ 180.5). The above functionalities accounted for six out of the 11 degrees of unsaturation, suggesting that 1 is pentacyclic. The HSQC, ^1^H–^1^H COSY, and HSQC-COSY spectra of 1 reveal three structural connectivities, namely I (H-2 to H-18, H-2 to H-4, and H-18 to both H_2_-19 and H_3_-20), II (H-6 to H_2_-7, H-11 to H_2_-12, and H-6 to H_2_-12), and III (H_2_-13 to H_2_-17), as is illustrated in Fig. 2.

**Table 1:** ^13^C and ^1^H NMR spectroscopic data for 1–3 in pyridine-*d*_5_ (125/500 MHz).

no.		1		2		3	
		^13^C	^1^H (J in Hz)	^13^C	^1^H (J in Hz)	^13^C	^1^H (J in Hz)
1		180.5 s		181.6 s		181.7 s	
2		46.1 d	2.37 m	45.8 d	2.27 m	45.5 d	2.15 m
3	a	35.9 t	2.03 m	28.9 t	1.92 m	27.7 t	1.87 m
3	b		2.75 m		2.55 m		2.91 m
4	a	76.6 d	5.51 dd (11.7, 3.6)	36.3 t	1.93 m	35.2 t	2.19 m
	b				2.16 m		2.27 m
5		51.0 s		45.9 s		45.2 s	
6		40.3 d	3.12 m	41.8 d	2.85 m	41.3 d	2.97 m
7	a	45.8 t	2.59 dd (13.3, 3.2)	46.0 t	2.56 dd (13.1, 3.3)	43.9 t	3.83 dd (13.3, 3.7)
	b		4.65 t (13.3)		4.66 t (13.1)		4.67 t (13.3)
8		134.1 s		136.4 s		126.7 s	
9		143.3 s		142.1 s		148.7 s	
10		134.6 s		134.6 s		168.2 s	
11		120.9 d	5.35 m	120.3 d	5.32 br d (5.4)	27.7 t	2.37 m
							2.61 m
12	a	33.2 t	2.06 m	32.9 t	1.99 m	25.9 t	1.68 m
	b		2.72 m		2.63 m		2.22 m
13	a	42.4 t	3.19 d (14.5)	42.8 t	2.90 d (15.2)	134.2 d	7.03 s
	b		3.76 m		3.72 m		
14		47.2 d	3.27 t (7.9)	46.6 d	3.21 dd (7.6, 7.3)	119.4 s	
15		54.3 d	3.69 m	54.3 d	3.67 m	160.2 s	
16	a	27.7 t	1.09 m	27.8 t	1.11 m	43.6 t	2.74 m, 2H
	b		1.74 m		1.73 m		
17	a	38.5 t	2.41 m	38.4 t	2.41 dd (14.4, 6.8)	25.5 t	2.77 m
	b		2.57 m		2.59 m		2.84 m
18		28.1 d	2.33 m	28.0 d	2.30 m	27.9 d	2.29 m
19	a	51.4 t	3.16 m, 2H	51.8 t	3.13 t (9.5)	51.6 t	3.17 t (9.7)
	b				3.16 t (9.5)		3.22 t (9.7)
20		12.5 q	1.25 d (6.7)	12.6 q	0.97 d (6.7)	12.4 q	1.00 d (7.0)
21	a	66.4 t	4.06 d (11.6)	73.0 t	4.13 d (11.0)	77.0 t	3.91 d (11.0)
	b		5.03 d (11.6)		4.40 d (11.0)		4.47 d (11.0)
22		173.9 s		173.9 s		164.9 s	
23		50.4 q	3.59 s	50.3 q	3.59 s	50.3 q	3.75 s
4-OAc		168.9 s					
		20.1 q	2.05 s				
21-OAc		170.5 s		170.8 s		170.2 s	
		20.4 q	2.07 s	20.4 q	2.15 s	20.3 q	2.13 s

Subsequent HMBC spectral analysis revealed where these three structural fragments are linked. The C-1 carbonyl (*δ*_C_ 180.5) and C-19 (*δ*_C_ 51.4, *δ*_H_ 3.16, m, 2 H) of fragment I were speculated to be due to a γ-lactam ring, which was verified by the observed HMBC-derived H_2_-3/C-1 and H_2_-19/C-1 correlations. HMBC correlations between H_2_-21 and C-4, C-5, C-6, and C-8, and between H_2_-7 and C-1 and C-19 linked fragment I to II, resulting in an amide-bridged bicyclic system (C-1 to C-7 and the N atom, and C-18 to C-19). The link between the methyl carboxylate and C-14 in fragment III was made on the basis of the HMBC correlations between both H-14 and H_3_-23, and C-22, while C-13, C-15, and C-17 are attached to C-8, C-9, and C-10 of the conjugated diene on the basis of the HMBC correlations between H-14 and C-8 and C-9, and between H-11 and C-9 and C-17. The acetoxy groups at C-4 and C-21 were assigned on the basis of HMBC correlations between H-4 and H_2_-21 and the respective acetyl carbonyls at *δ*_C_ 168.9 and 170.5. Hence, the planar structure of 1 was assigned to be a fused pentacyclic ring with a γ-lactam functionality (Fig. 2).

**Figure 3:**
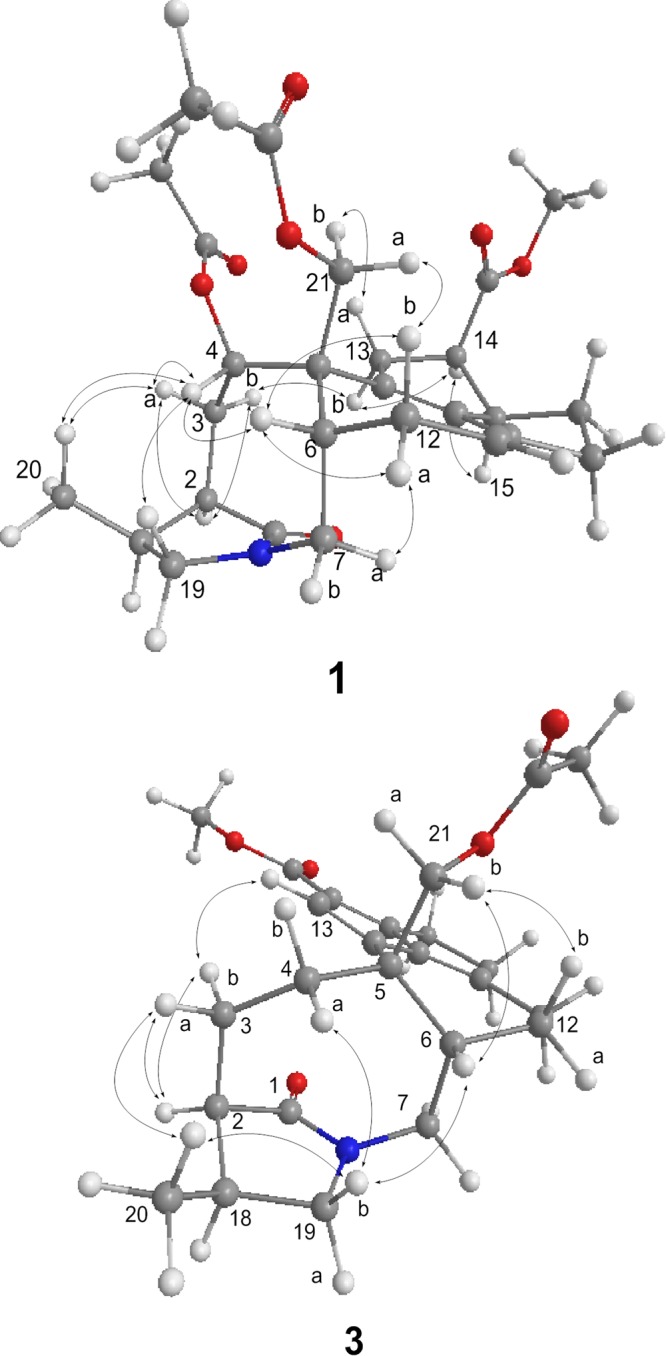
Selected NOESY correlations in 1 and 3.

The relative configuration of 1 was deduced through NOE-correlation analysis, as indicated in the Chem3D drawing (Fig. 3); H-4/H-19, H-4/H-6, H-4/H_3_-20, H-21a/H-12b, and H-6/H_2_-12 NOE correlations indicate that H_2_-21, H-6, H_3_-20, and H-4 are cofacial and were arbitrarily assigned to be β-oriented. In addition, the NOE spectrum reveals a correlation between H-2 and H_2_-3, in addition to a correlation between H-3a and H_3_-20, implying that H-2 is positioned between H_2_-3, but opposite to H_3_-20 (Fig. 3). In addition, H-13a/H-21b, H-13b/H-14, and H-14/H-15 correlations suggest that both H-14 and H-15 are α-oriented.

High-resolution electrospray-ionization mass spectrometry (HRESIMS) revealed that glaulactam B (2) has the molecular formula C_25_H_33_NO_5_, the mass of which is less than that of 1 by one CH_2_CO_2_ unit. In addition, the ^13^C and ^1^H NMR data for 2 are similar to those of 1; however, instead of possessing an acetoxy-bearing methine, as in 1, an sp^3^ methylene is present at C-4 (*δ*_C_ 36.3) in 2 (Table 1). This was confirmed by the HMBC correlations between H_2_-21 and C-4, C-5, C-6, and C-8 (Fig. 2). The planar structure of 2 (Fig. 2) was established following ^1^H-^1^H COSY, HSQC-TOCSY, and HMBC spectral analyses. With the exception of C-4, the same relative configurations were assigned to 1 and 2 on the basis of detailed NOE-correlation analyses (Supplementary Fig. S4).

Glaulactam C (3) was subjected to HRESIMS, which suggested that its molecular formula was C_25_H_31_NO_5,_ which contains two less hydrogen atoms than 2. IR absorptions at 1735, 1701, 1687, and 1641 cm^−1^ indicate the presence of carbonyl and alkene functionalities. The ^1^H and ^13^C NMR spectra of 3 reveal the presence of characteristic signals that are attributable to a similar γ-lactam ring (*δ*_C_ 181.7, 45.5, 27.9, 51.6, and 12.4), a 21-acetoxy moiety (*δ*_C_ 77.0, 170.2, and 20.3), and a 14-methyl carboxylate (*δ*_C_ 164.9, 50.3) functionality as was observed for 2. Moreover, the presence of six olefinic-carbon signals (*δ*_C_ 119.4, 126.7, 134.2, 148.7, 160.2, and 168.2; *δ*_H_ 7.03) and an upfield-shifted carboxylate carbon (*δ*_C_ 164.9) that is correlated to the methyl protons (*δ*_H_ 3.75) in the HMBC spectrum, suggest that the carboxylate is conjugated to the three alkene units (Fig. 2). The HMBC correlations between H-13 and C-8, C-9, C-14, and C-15, and between H_2_-16 and C-9 and C-10, as well as H_2_-11 and C-9, C-10, and C-17, led to the assignment of a fulvene moiety, as illustrated in Fig. 2. The planar structure of 3 was confirmed by further HSQC-TOCSY spin-system analyses (H-6 to H_2_-7, H-11 to H_2_-12, and H-6 to H_2_-12) and HMBC correlations between H_2_-21 and C-4, C-5, C-6, and C-8. The H-21b/H-6, H-19b/H_3_-20, and H-19b/H-6 NOE correlations imply that H-6, H_2_-21, and H_3_-20 are oriented on the same face of the molecule. Moreover, H_3_-20/H-3a, H-2/H-3a, and H-2/H-3b correlations suggest that H-2 is positioned opposite to H_3_-20 (Fig. 3).

**Figure 4:**
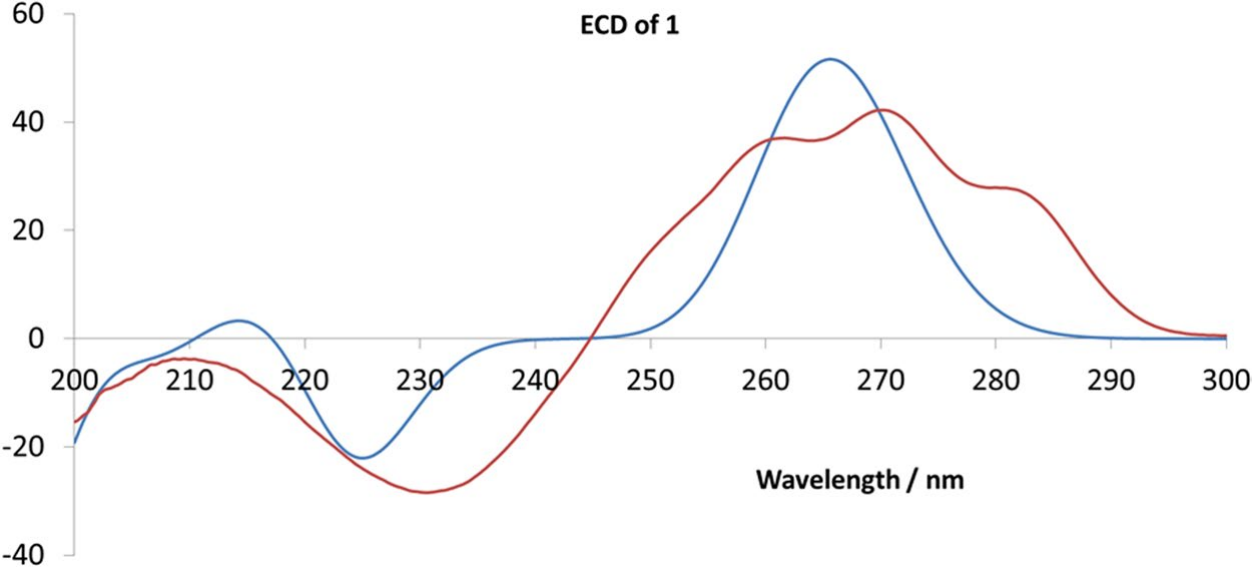
Experimental (red) and calculated (blue) ECD spectra of 1.

The absolute configurations of compounds 1–3 were determined by comparing the experimental electronic circular dichroism (ECD) spectra to those calculated theoretically. Compounds 1–3 were subjected to standard conformational analysis as implemented in the Confab program^13^. The generated lowest-energy structures following further B3LYP/6–31G(d) optimizations were then used to calculate ECD spectra by time-dependent density functional theory (TDDFT) at the TD-CAM-B3LYP/def2TZVP level. All calculations were performed using the Gaussian 09 Rev. D program package using the “ultrafine grid” option, *Integral (Grid* = *UltraFine)*, with solvent effects accounted for using the IEFPCM method^14^. The ECD spectrum of each compound 1–3 was finally generated as the Boltzmann-weighted sum of the spectra generated for the various conformers in each case, which resulted in the establishment of the absolute configurations of glaulactams A–C were, as illustrated by structures 1–3, since the calculated spectra are in good agreement with those acquired experimentally (Fig. 4; Supplementary Fig. S5).

**Figure 5:**
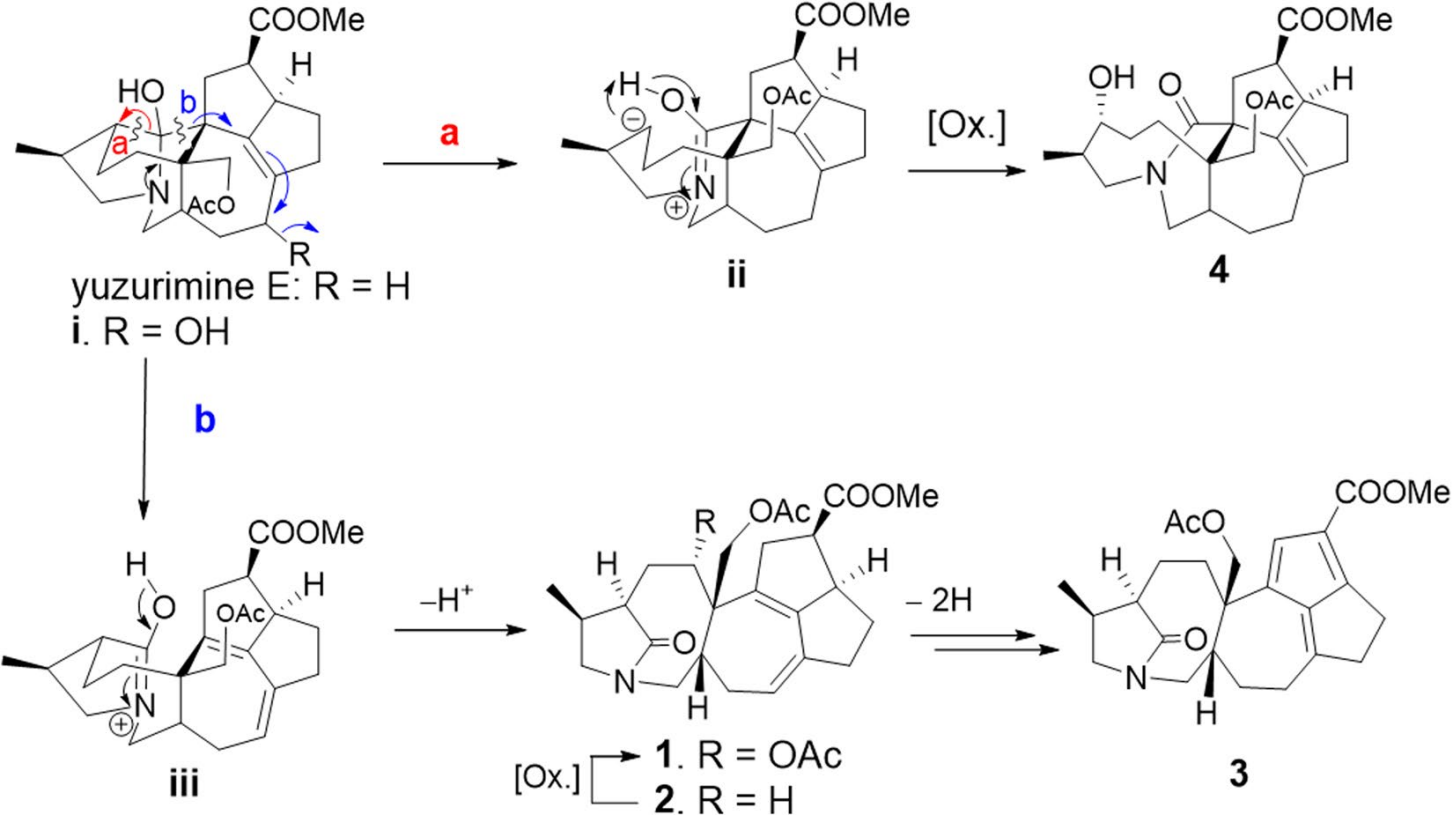
Plausible biogenetic pathways for 1–4.

It is possible to describe the biosynthetic origins of 1–4 on the basis of the lactam formation mechanism of hemiaminals, the structure of yuzurimine E^15^ and a proposed compound i. In the proposed mechanism (Fig. 5), oxidative cleavage of the C-1–C-2 bond in yuzurimine E via pathway *a*^16^ yields intermediate ii, which, after oxidation at C-2, gives 4. Similarly, C-1–C-8 bond cleavage via pathway *b* results in the formation of intermediate iii; subsequent deprotonation of the iminol affords lactam 2, which then undergoes oxidation at C-4 and acetylation to yield 1. Double-bond migration and dehydrogenation then result in the transformation of 2 into fulvene 3.

**Figure 6:**
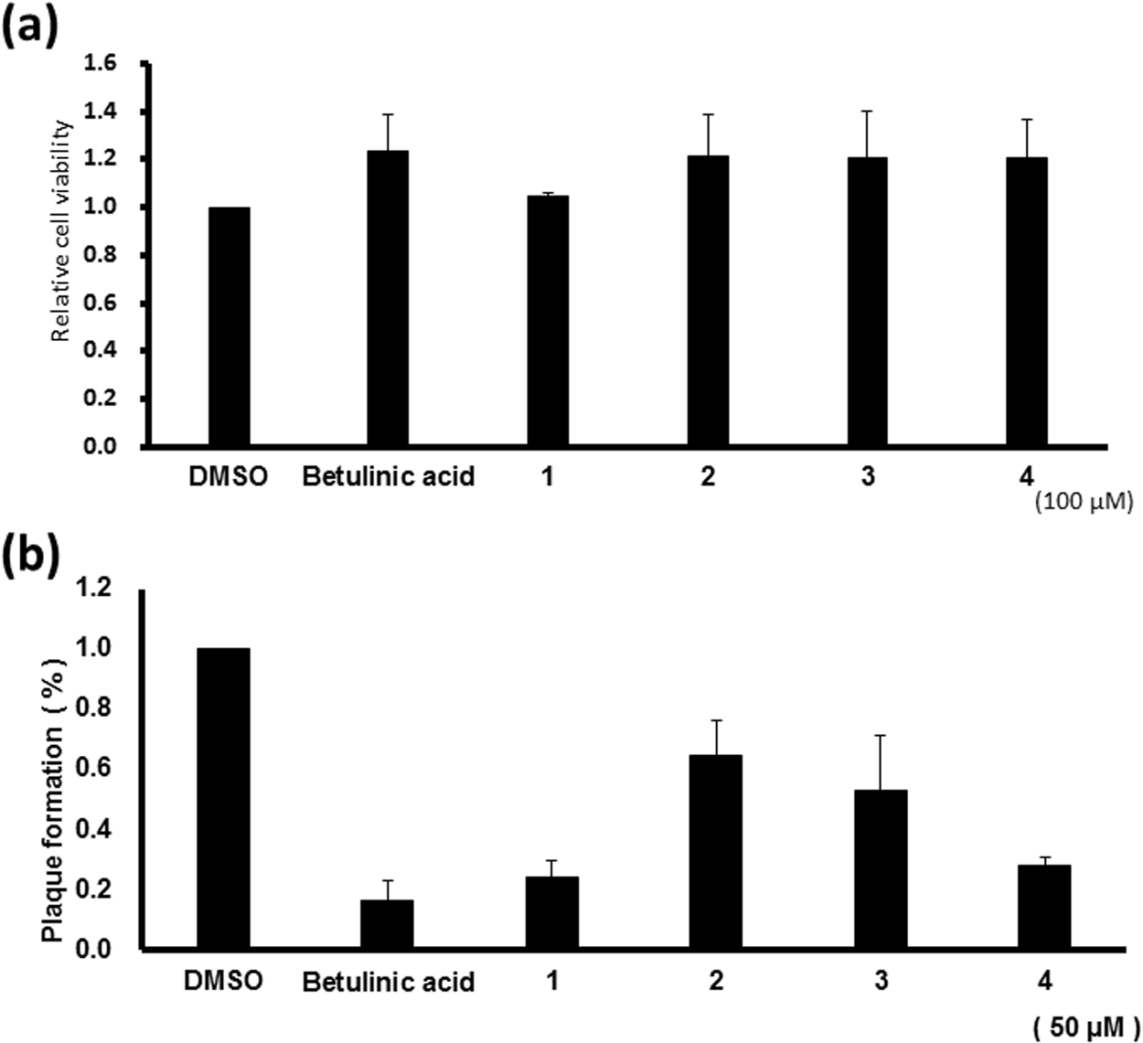
Cytotoxic (**a**) and anti-H1N1(**b**) activities of compounds 1–4. Betulinic acid was used as the positive control.

**Figure 7:**
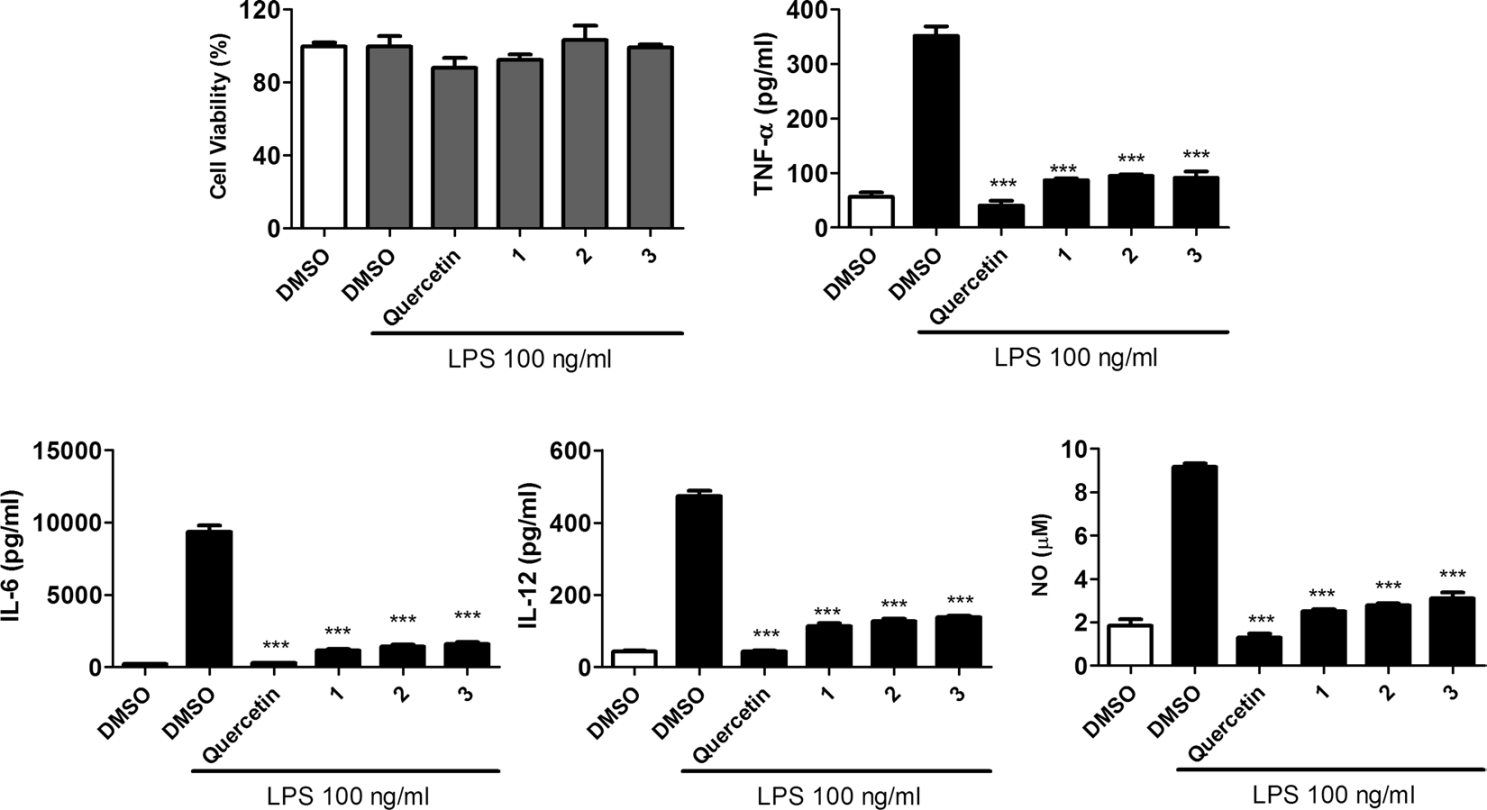
(**a**) Cytotoxicities toward murine bone marrow-derived DCs in the presence of 100 ng/mL LPS for 24 h using the CCK-8 cell-counting assay. (**b**) TNF-α, (**c**) IL-6, and (**d**) IL-12 p70 cytokine levels determined by ELISA, and (**e**) NO determined using the Griess reagent. Data are expressed as means ± standard deviation (n = 3). Compounds 1–3 were used at 50 μg/mL and quercetin, the positive control, was used at 50 μM.

In preliminary biological screening, the extract of *D*. *glaucescens* exhibited antiviral activity against the influenza virus and was immunosuppressive in lipopolysaccharide (LPS)-stimulated murine dendritic cells (DCs). Compounds 1–4 were tested for their anti-influenza virus (i.e., anti-H1N1) activities in Madin-Darby canine kidney (MDCK) cells using the plaque assay with betulinic acid as the positive control^17^. Cytotoxicity testing revealed that the isolates were not toxic to uninfected host MDCK cells at a concentration of 100 μM (Fig. 6a). However, at a concentration of 50 μM, compounds 1 and 4 were found to substantially inhibit plaque formation of MDCK cells by H1N1 virus infection, to values of 24.4% and 28.0%, respectively. Although less effective under the same treatment conditions, compounds 2 and 3 were found to moderately inhibit plaque formation (69.1% and 63.5%, respectively) (Fig. 6b). In addition, compounds 1–3 were also evaluated for their immunosuppressive activities. Mouse bone-marrow DCs were treated with compounds 1–3, and the immunosuppressive agent quercetin was used as the positive control^18^. In preliminary studies, compounds 1–3 (50 μg/mL) and quercetin (50 μM) were found to have no significant cytotoxic effects on murine DCs in the presence of LPS (100 ng/mL) (Fig. 7). However, subsequent experiments revealed that compounds 1–3 significantly suppress the levels of tumor necrosis factor-α (TNF-α), interleukin-6 (IL-6), IL-12p70, and nitric oxide (NO) in LPS-stimulated murine DCs (Fig. 7). These results confirm that the immunosuppressive properties of compounds 1–3 are not due to their cytotoxicities in DCs, and that the observed effects are similar to that induced by quercetin.

In summary, IXC and NMR fingerprinting were used to identify and isolate three novel daphniphyllum alkaloids, whose anti-influenza and immunosuppressive activities were then explored. The method described herein can be implemented as a convenient alternative to existing methods commonly employed to extract unique compounds from a complex array of natural products.

## Methods

### Ethical Statement

The Institutional Animal Care and Use Committee (IACUC) of National Chung Hsing University approved the experimental procedures (approved protocol no. NCHU-IACUC-104-027). The methods were performed in accordance with the approved guidelines.

### General experimental procedures

Optical rotations were measured on a JASCO P2000 digital polarimeter and IR spectra were acquired on a Shimadzu IR Prestige-21 FT-IR spectrometer. NMR spectra, in pyridine-*d*_5_, were recorded on a 500 MHz Avance III spectrometer (Bruker, Rheinstetten, Germany). The ^1^H and ^13^C NMR chemical shifts were referenced to the solvent residual peaks at δ_H_ 7.58 and δ_C_ 135.5 for pyridine-*d*_5_. HRESIMS was performed on an LTQ Orbitrap XL mass spectrometer. Silica gel 60 (Merck, 230−400 mesh), Diaion HP-20 (Supelco™, Bellefonte, PA, USA), and RP-18 gel (LiChroprep 40–63 μm, Merck) were used for column chromatography. A Shimadzu LC-20AT pump and Shimadzu SPD-M20A diode array detector (Shimadzu Inc., Kyoto, Japan) equipped with an Inertsil ODS-3 column (5 µm, 250 × 10 mm, GL Science Inc., Tokyo, Japan) were used for HPLC. Preparative medium-pressure liquid chromatography (MPLC) was performed on an Interchim PuriFlash XS 420 chromatographic system.

### Plant Material

The leaves of *D*. *glaucescens* subsp. *oldhamii* were collected in Yilan County, Taiwan in October 2014. The plant was identified by Dr. S.-Y. Hwang. A voucher specimen (specimen no. DG-Chao004) was deposited at the Chinese Medicine Research and Development Center, China Medical University Hospital.

### Extraction and Isolation

Air-dried leaves of *D*. *glaucescens* (10.38 kg) were exhaustively minced and extracted with MeOH (4 × 20 L). The solvent was concentrated, and 20% water was added to yield an aqueous suspension, to which hexane (4 × 2 L) was added to removed lipids and chlorophylls. The methanolic solution was passed through a strong cation-exchange resin (Dowex 50WX4) to adsorb alkaloids (Supplementary Fig. S6). The collected eluent, which contained nonalkaloid constituents (1530 g), was fractionated by column chromatography (CC; Diaion HP-20: column size: 12.0 × 25.0 cm; successively eluted with 10 L of 30%, 60%, 80%, and 100% MeOH in H_2_O) to yield four fractions. Fraction 4 (24.95 g), which was eluted with 100% MeOH, was fractionated by silica-gel CC (column size: 6.0 × 15.0 cm; eluted with 9:1 to 0:1 hexane:EtOAc) to yield 26 subfractions (4A–4W). Subfraction 4V (203 mg), which exhibited ^1^H NMR signals characteristic of daphniphyllum alkaloids (i.e., few methyl signals in the 1–2 ppm region, aliphatic signals at 2–3 ppm, and C*H*-N signals between 3 and 4 ppm; see Supplementary Fig. S6), were selected for further purification by MPLC (PuriFlash Column, 30 µm, PF-30C18XS/55G; eluted with 70% to 100% MeOH in H_2_O) to yield 1 (4.2 mg) and 2 (29.2 mg). Subfraction 4 W (288 mg) was fractionated by MPLC (PuriFlash Column, 30 µm, PF-30C18XS/55G; 60% to 100% MeOH in H_2_O) to give six subfractions (4W1 to 4W6), of which 4W6 was found to possess signals characteristic of daphniphyllum alkaloids. Compound 4 (2.8 mg) was isolated from subfraction 4W6 by HPLC (65% MeOH in H_2_O). Subfraction 4Z (2.0 g) was chromatographed on silica gel (1:1 to 0:1 hexane:EtOAc), followed by MPLC (PuriFlash Column, 30 µm, PF-30C18XS/55G; 70% to 100% MeOH in H_2_O) to yield subfraction 4Z2A; the ^1^H NMR signals of subfraction 4Z2A were found to be characteristic of daphniphyllum alkaloids. Further purification of subfraction 4Z2A by HPLC (65% MeOH in H_2_O) yielded compound 3 (3.0 mg).

Glaulactam A (1): colorless oil; [α] + 102 (*c* 0.42, MeOH); ECD (EtOH) *λ*_max_ (∆*ε*) 231 (−8.61), 270 (+12.81); UV (MeOH) *λ*_max_ (log *ε*) 260 (3.74), 270 (3.81), 280 (3.71) nm; IR (KBr) *v*_max_ 2949, 2933, 1736, 1730, 1712, 1695, 1433, 1371, 1247, 1224, 1195, 1168, 1028, and 754 cm^−1^; ^13^C and ^1^H NMR data, see Table 1; ESIMS *m*/*z* 486 [M + H]^+^, 508 [M + Na]^+^; HRESIMS *m*/*z* 486.2494 [M + H]^+^ (calcd. for C_27_H_36_NO_7_, 486.2486).

Glaulactam B (2): colorless oil; [α] + 146 (*c* 0.58, MeOH); ECD (EtOH) *λ*_max_ (∆*ε*) 229 (−7.33), 270 (+5.40); UV (MeOH) *λ*_max_ (log *ε*) 261 (3.67), 271 (3.74), 280 (3.63) nm; IR (KBr) *v*_max_ 2949, 2931, 1734, 1699, 1685, 1436, 1375, 1363, 1244, 1193, 1166, 1028, and 752 cm^−1^; ^13^C and ^1^H NMR data, see Table 1; ESIMS *m*/*z* 428 [M + H]^+^, 450 [M + Na]^+^; HRESIMS *m*/*z* 428.2436 [M + H]^+^ (calcd. for C_25_H_34_NO_5_, 428.2431).

Glaulactam C (3): colorless oil; [α] + 70 (*c* 0.30, MeOH); ECD (EtOH) *λ*_max_ (∆*ε*) 226 (−9.02), 287 (+3.21); UV (MeOH) *λ*_max_ (log *ε*) 245 (4.07), 288 (3.91) nm; IR (KBr) *v*_max_ 2926, 1735, 1701, 1687, 1641, 1597, 1436, 1365, 1284, 1234, 1195, 1172, 1118, 1062, 1028, and 754 cm^−1^; ^13^C and ^1^H NMR data, see Table 1; ESIMS *m*/*z* 448 [M + Na]^+^; HRESIMS *m*/*z* 448.2090 [M + Na]^+^ (calcd. for C_25_H_31_NO_5_Na, 448.2094).

### Cell culture and cytotoxicity assay

Madin-Darby canine kidney (MDCK) cells, (obtained from Dr. Lin, JH, Centers for Disease Control, Taipei, Taiwan) were cultured in Dulbecco’s Modified Eagle’s Medium (DMEM, Hyclone, GE Healthcare Life science) supplemented with 10% heat-inactivated fetal bovine serum (FBS, Hyclone, GE Healthcare Life science) and maintained at 37 °C in a 5% CO_2_ atmosphere. For the cytotoxicity assay, MDCK cells were seeded in 96-well microplates (1 × 10^4^ cells/well), and cultured for 24 h. Culture media were then replaced with media containing each of the indicated compounds for an additional 72 h. Cell viability was determined by the 3-(4,5-dimethylthiazol-2-yl)-5-(3- carboxymethoxyphenyl)-2-(4-sulfophenyl)-2*H* tetrazolium (MTS) assay kit purchased from Promega (Madison, WI).

### Plaque-reduction assay

Monolayer MDCK cells were seeded in six-well plates (5 × 10^5^ cells/well) for 24 h. The influenza virus A/WSN/33(H1N1) (100 plaque-forming units (PFU) per well) was mixed with each of the indicated compounds for 30 min at room temperature. The mixtures were subsequently adsorbed to the preseeded cells for 1 h at 37 °C. After removal of the medium, the cells were washed with PBS (three times) and then overlaid with 0.3% agarose containing the indicated compounds for an additional 48 h at 37 °C, after which the cells were fixed with 10% formaldehyde for 1 h. Viral plaques were counted by staining with 0.5% crystal violet.

### Mice and the preparation of bone marrow-derived murine dendritic cell (DC)

According to the published method^19^, the murine bone-marrow-derived DCs were prepared from female C57BL/6 mice housed under controlled-temperature (22 ± 2 °C) and humidity (45–65%) conditions, with a 12-h light/dark cycle and free access to food and water. The animals were treated according to the requirements of the Institutional Animal Care and Use Committee of National Chung-Hsing University.

### Cell viability assay

DC-cell viability was measured using a cell-counting-kit assay (CCK-8; Dojindo Molecular Technologies, Inc., Kumamoto, Japan)^20^, at an absorbance wavelength of 450 nm.

### Cytokine and nitric oxide (NO) assay

After centrifugation at 1000 × *g* for 15 min at 4 °C, the levels of tumor necrosis factor (TNF)-α (cat. no. 900-K54; PeproTech, Inc., London, UK), interleukin (IL)-6 (cat. no. 900-K50; PeproTech, Inc., London, UK), and IL-12p70 (cat. no. 900-K97; PeproTech, Inc., London, UK) in the culture supernatants were determined using murine ELISA kits for the respective cytokine according to the manufacturer’s protocol. The levels of NO were measured indirectly by determining the concentration of NO^2-^ using a spectrophotometric assay based on the Griess reaction.

### Computational details

Since different conformers of a specific stereochemical configuration can give different ECD spectra, it is critical to identify all relevant conformers in order to accurately predict the ECD spectrum. Therefore, standard conformational analyses were performed using the Confab program^13^. All optimized conformations were in a 10 kcal/mol energy window, with a root mean square (RMS) step-size of 0.2 Å. These conformers were then re-optimized at the B3LYP/6-31G(d) level of theory and verified to be true minima on the potential energy surface by frequency analyses. Then resulting geometries were subsequently used for three single-point calculations. CAM-B3LYP/TZVP^21^ calculations in the vacuum state were carried out in order to obtain converged wavefunctions for the ground states, which were used in the next two calculations. Energies were calculated at the M062x/Def2TZVP level with ethanol as the solvent, as this method provides more precise energies for conformational ordering^22^. The series of conformers was restricted by removing duplicates (RMSD < 0.2) and conformations outside of a 4 kcal/mol energy window, based on the Gibbs free energy at the M062x/Def2TZVP//B3LYP/6-31G(d) level in ethanol. The resulting structures were used in TDDFT ECD calculations, in ethanol as the solvent, at the TD-CAM-B3LYP/def2TZVP level by considering the 100 lowest-energy states. All calculation were performed using the Gaussian 09 program package with the “ultrafine grid” option, *Integral (Grid* = *UltraFine)*, and all solvent effects were accounted for using the IEFPCM method^14,23^. Finally, the overall ECD spectra were combined following Boltzmann weighting on the basis of the Gibbs free energies of the corresponding conformer calculated at the M062x/Def2TZVP//B3LYP/6-31G (d) level. All ECD spectra were processed with SpecDis and simulated by Gaussian functions with bandwidths (σ) of 0.16 eV and by considering velocity representations^24^.

## Electronic supplementary material

supplementary information
