# Supplementary material for: Glaulactams A–C, daphniphyllum alkaloids from Daphniphyllum glaucescens
Source: Sci Rep. 2018 Oct 18;8:15417. doi: 10.1038/s41598-018-33748-6 (PMC6193994; doi:10.1038/s41598-018-33748-6)
Supplement: Supplementary file 1 — supplementary information [file 41598_2018_33748_MOESM1_ESM.pdf]

Glaulactams A–C, daphniphyllum alkaloids from *Daphniphyllum glaucescens*

Chih-Hua Chao,* Ju-Chien Cheng, Théo P. Gonçalves, Kuo-Wei Huang, Chi-Chien Lin, Hui-Chi Huang, Syh-Yuan Hwang, and Yang-Chang Wu*

*Correspondence and requests for materials should be addressed to C.-H.C. (e-mail: chaochihhua@hotmail.com) and Y.-C.W. (e-mail: yachwu@kmu.edu.tw)

Figure S1-1 ^{1}H NMR spectrum (500 MHz) of compound **1** in pyridine-*d*5. S1
Figure S1-2. ^{13}C NMR spectrum (125 MHz) of compound **1** in pyridine-*d*5. S2
Figure S1-3. ^{1}H-^{1}H COSY spectrum of compound **1** in pyridine-*d*5. S3
Figure S1-4. NOESY spectrum of compound **1** in pyridine-*d*5. S4
Figure S1-5. HSQC spectrum of compound **1** in pyridine-*d*5. S5
Figure S1-6. HMBC spectrum of compound **1** in pyridine-*d*5. S6
Figure S1-7. HSQC-TOCSY spectrum of compound **1** in pyridine-*d*5. S7
Figure S2-1. ^{1}H NMR spectrum (500 MHz) of compound **2** in pyridine-*d*5. S8
Figure S2-2. ^{13}C NMR spectrum (125 MHz) of compound **2** in pyridine-*d*5. S9
Figure S2-3. HSQC spectrum of compound **2** in pyridine-*d*5. S10
Figure S2-4. HMBC spectrum of compound **2** in pyridine-*d*5. S11
Figure S2-5. NOESY spectrum of compound **2** in pyridine-*d*5. S12
Figure S3-1. ^{1}H NMR spectrum (500 MHz) of compound **3** in pyridine-*d*5. S13
Figure S3-2. ^{13}C NMR spectrum (125 MHz) of compound **3** in pyridine-*d*5. S14
Figure S3-3. HSQC spectrum of compound **3** in pyridine-*d*5. S15
Figure S3-4. HMBC spectrum of compound **3** in pyridine-*d*5. S16
Figure S3-5. NOESY spectrum of compound **3** in pyridine-*d*5. S17
Figure S4. Selected NOESY correlations of **2**. S18
Figure S5. Experimental (red) and calculated (blue) ECD spectra of **2** (left) and **3** (right). S19
Figure S6. Isolation process using ion exchange resin and NMR fingerprint method. S20

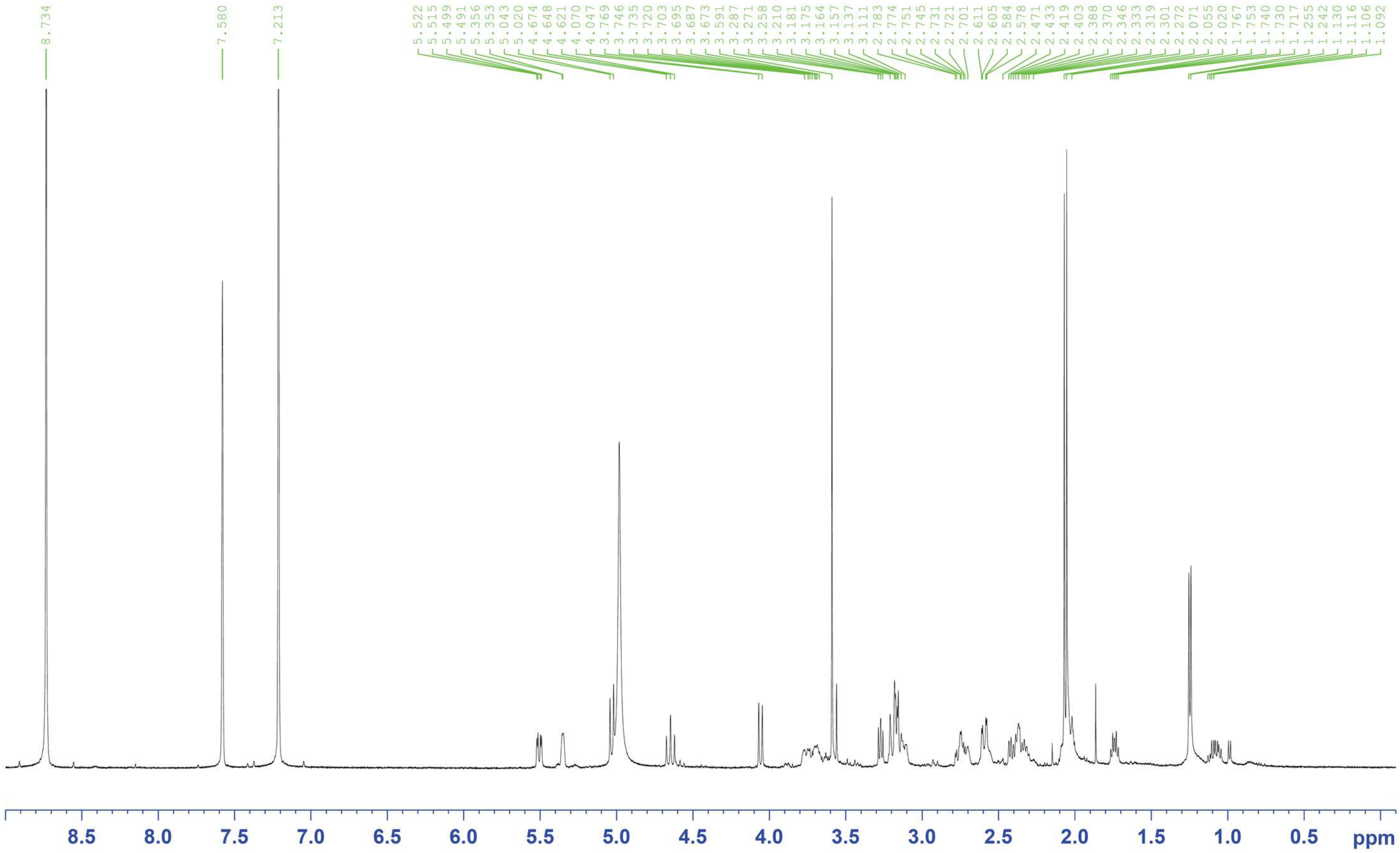

Figure S1-1 ^{1}H NMR spectrum (500 MHz) of compound **1** in pyridine-*d*5.

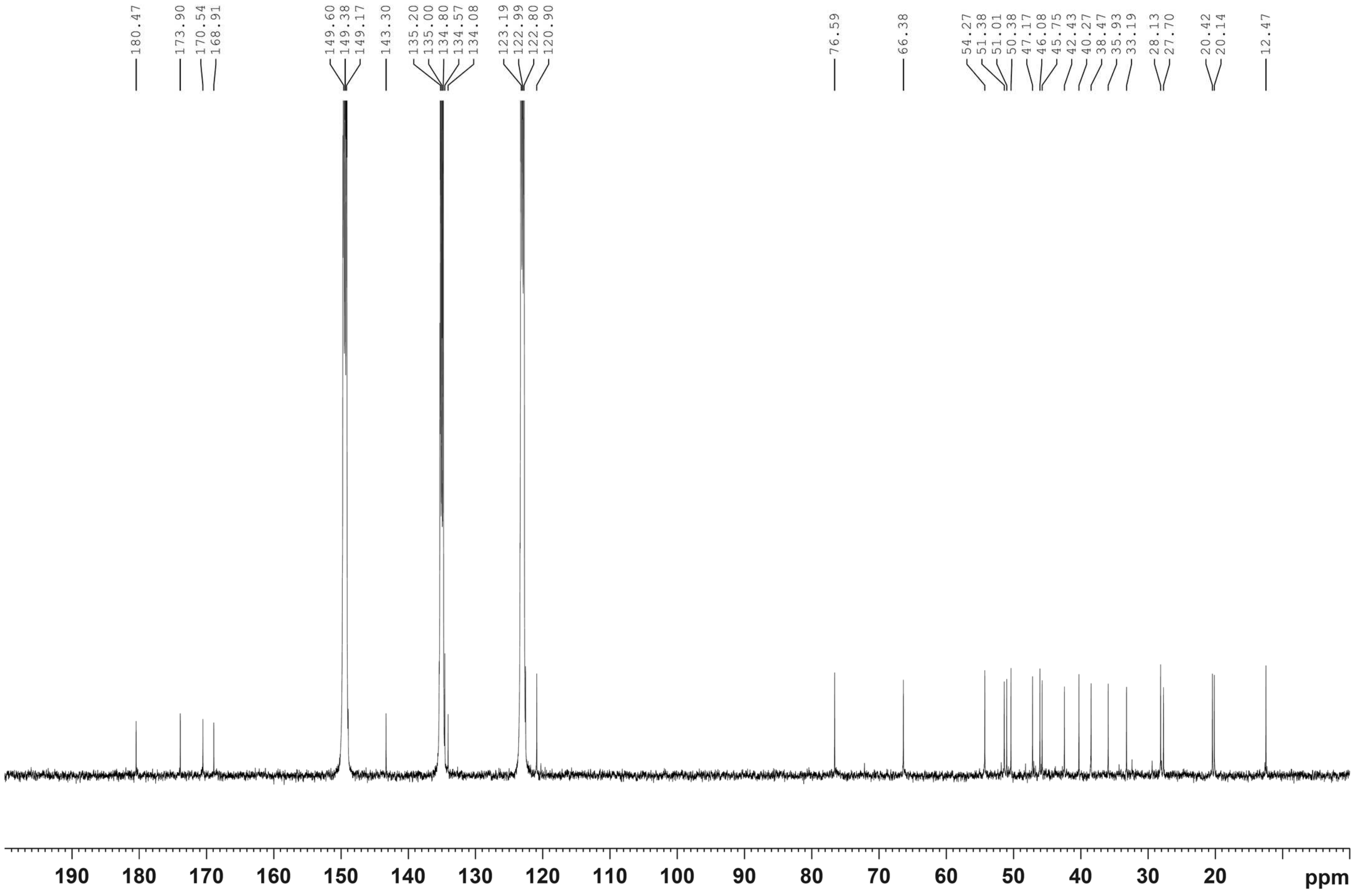

Figure S1-2. ^{13}C NMR spectrum (125 MHz) of compound **1** in pyridine-*d*5.

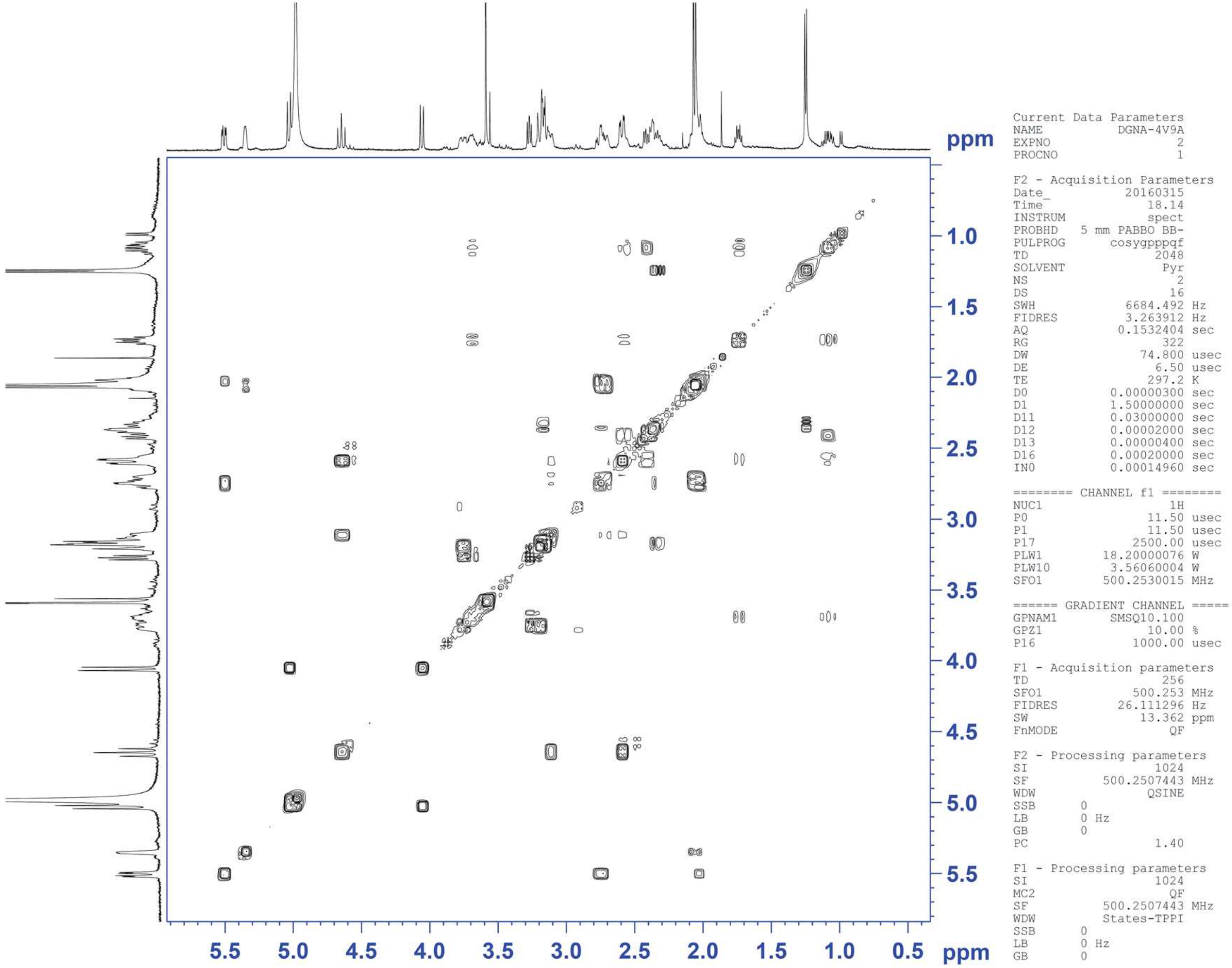

Figure S1-3. ^{1}H-^{1}H COSY spectrum of compound **1** in pyridine-*d*5.

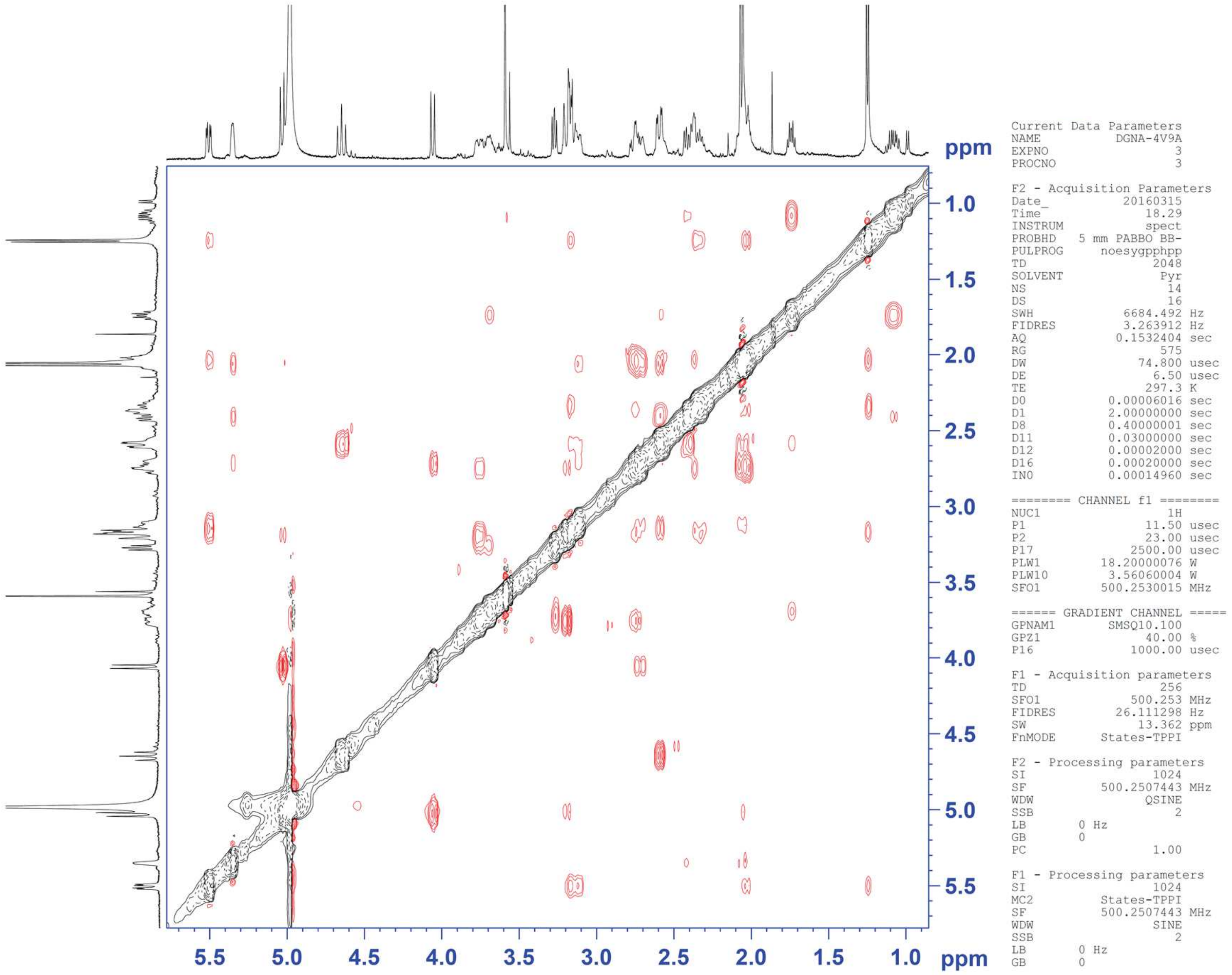

Figure S1-4. NOESY spectrum of compound **1** in pyridine-*d*5.

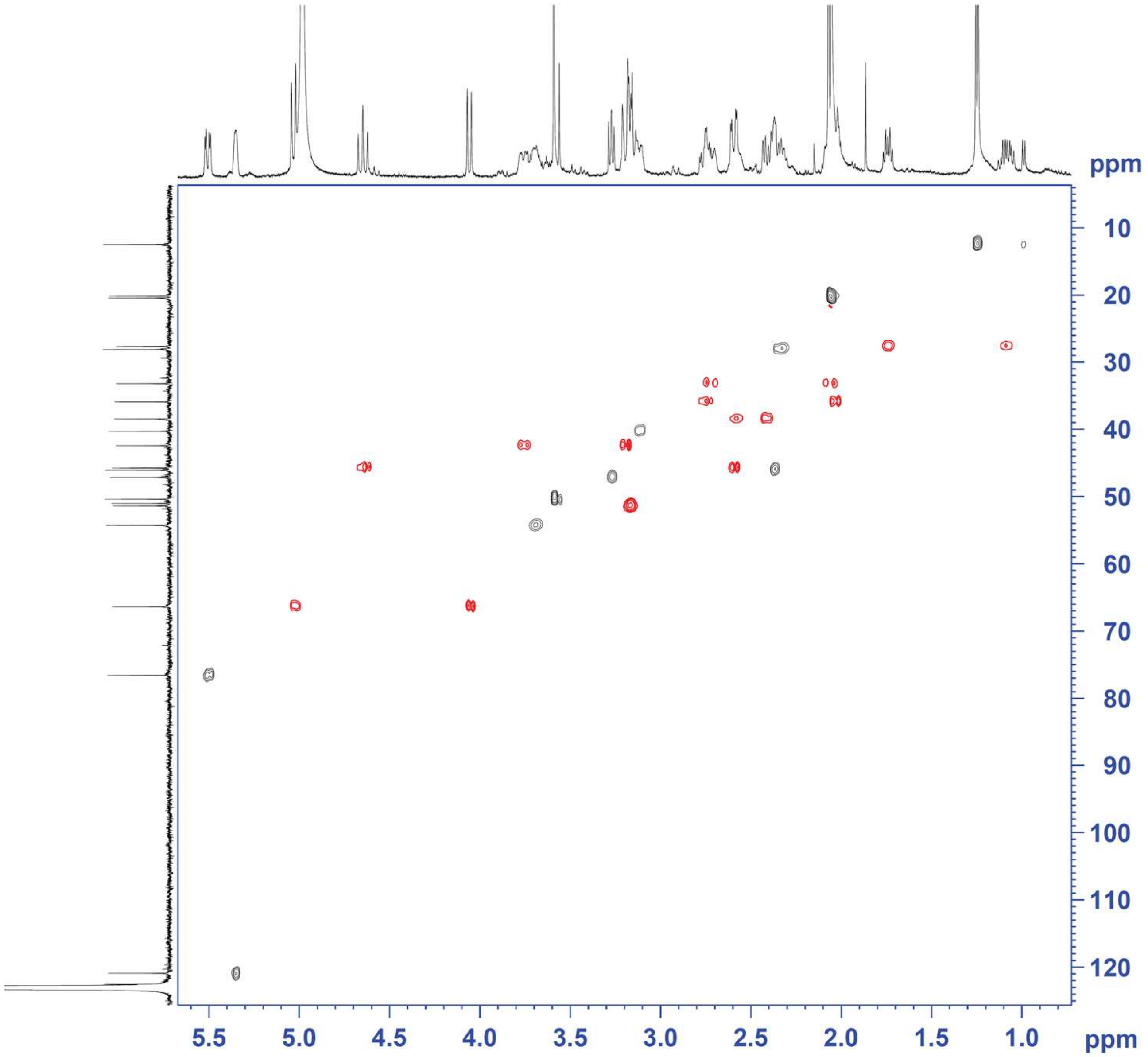

Figure S1-5. HSQC spectrum of compound **1** in pyridine-*d5*.

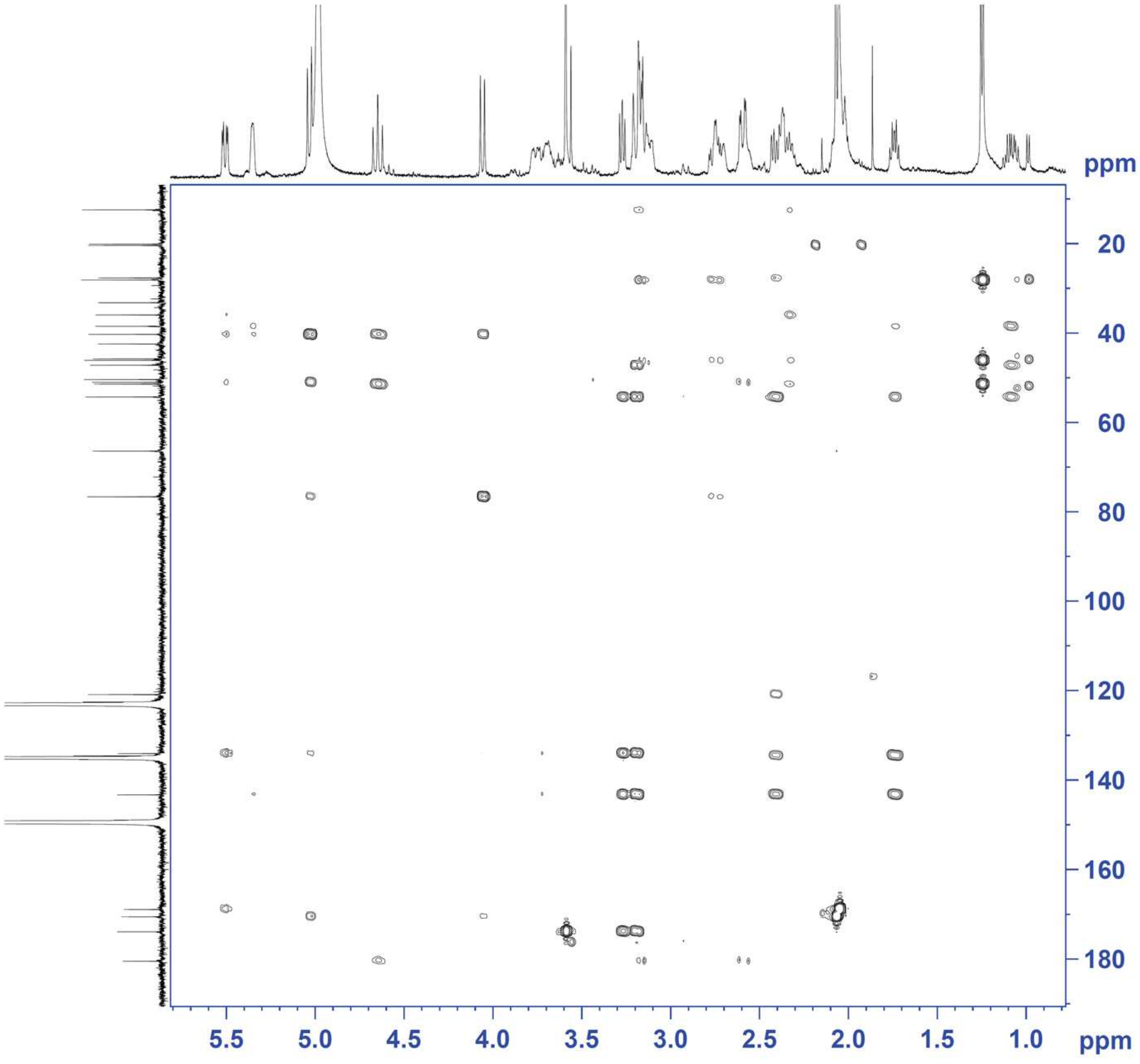

Figure S1-6. HMBC spectrum of compound **1** in pyridine-*d*5.

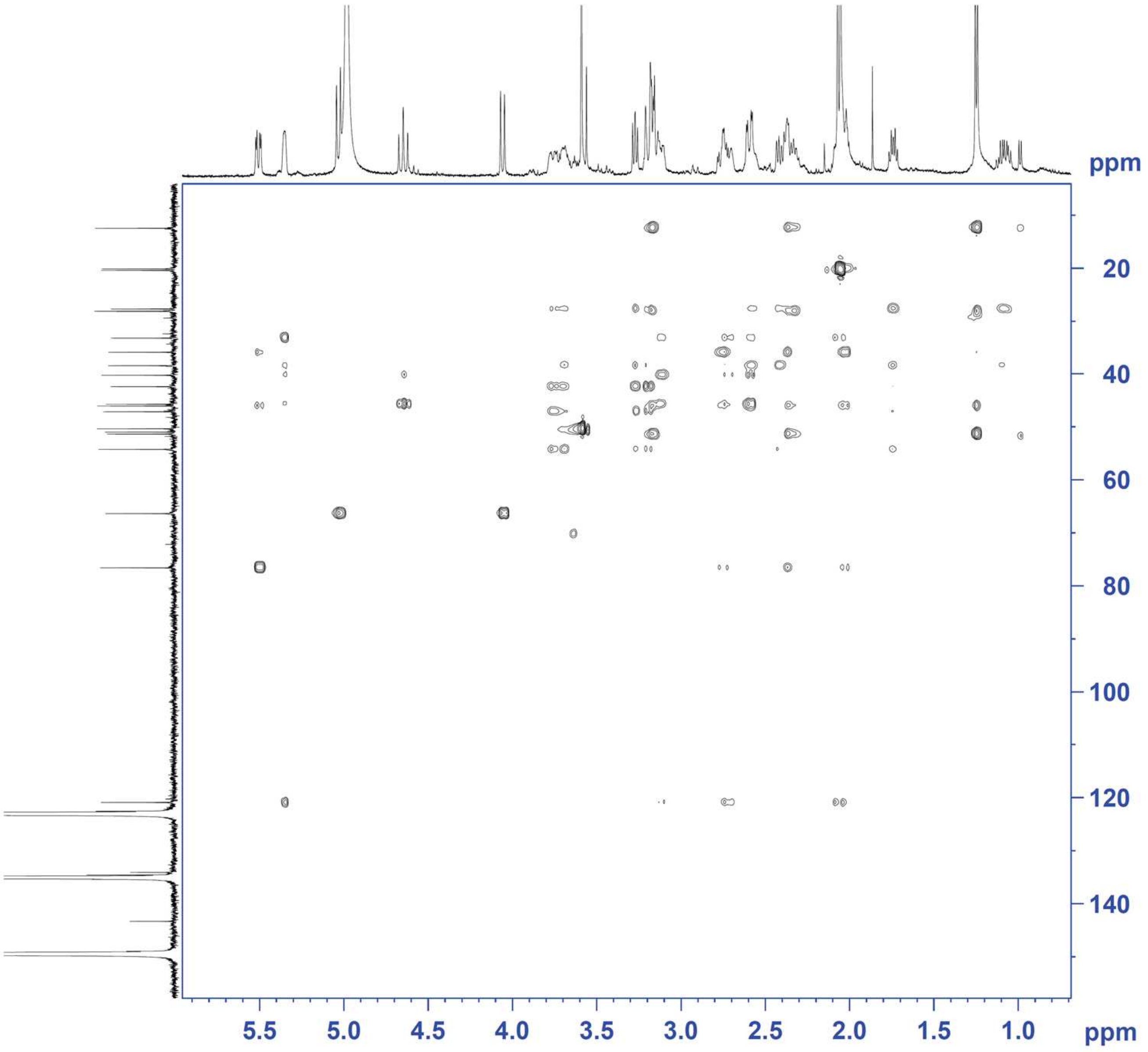

Figure S1-7. HSQC-TOCSY spectrum of compound **1** in pyridine-*d5*.

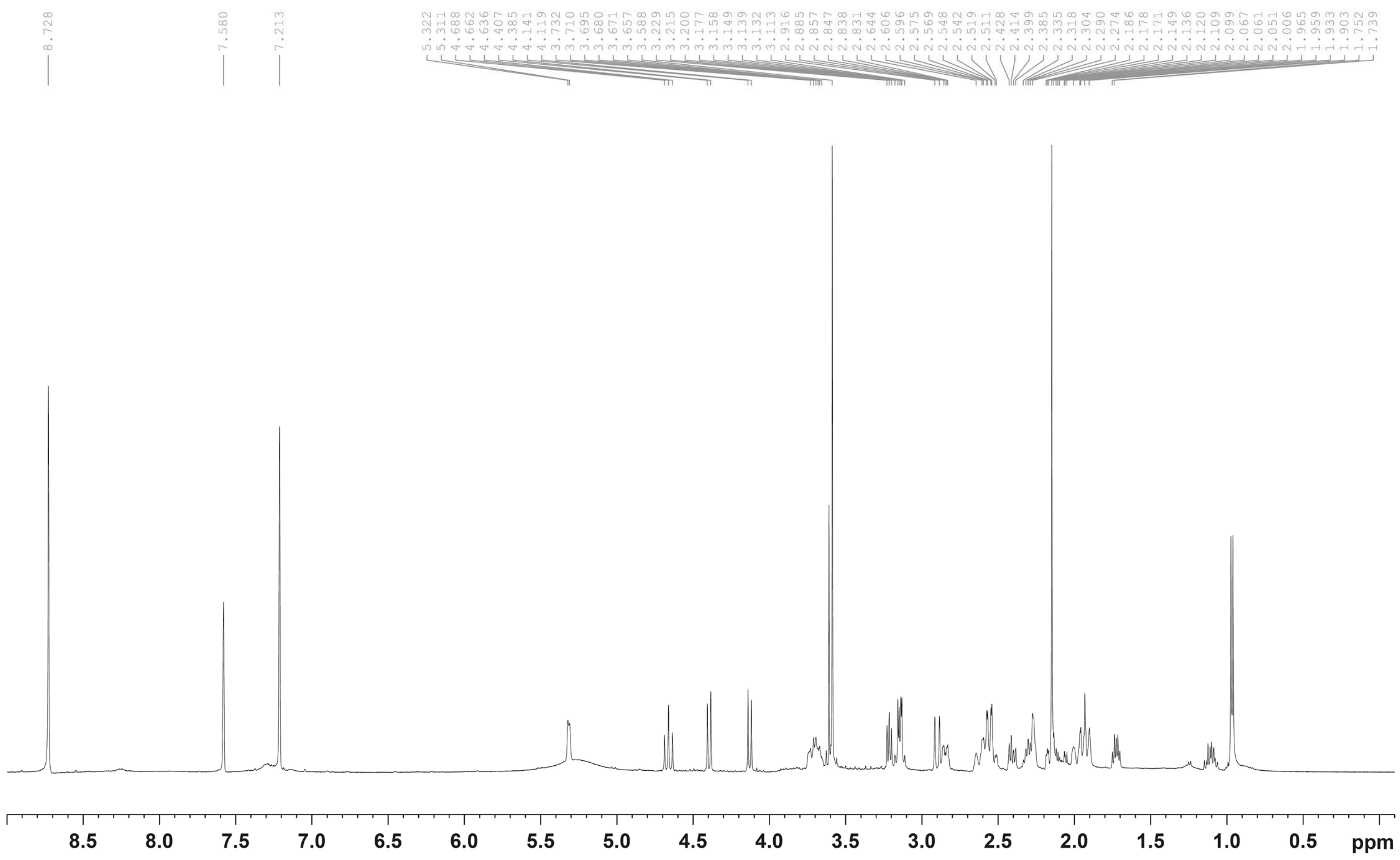

Figure S2-1. ^{1}H NMR spectrum (500 MHz) of compound **2** in pyridine-*d*5.

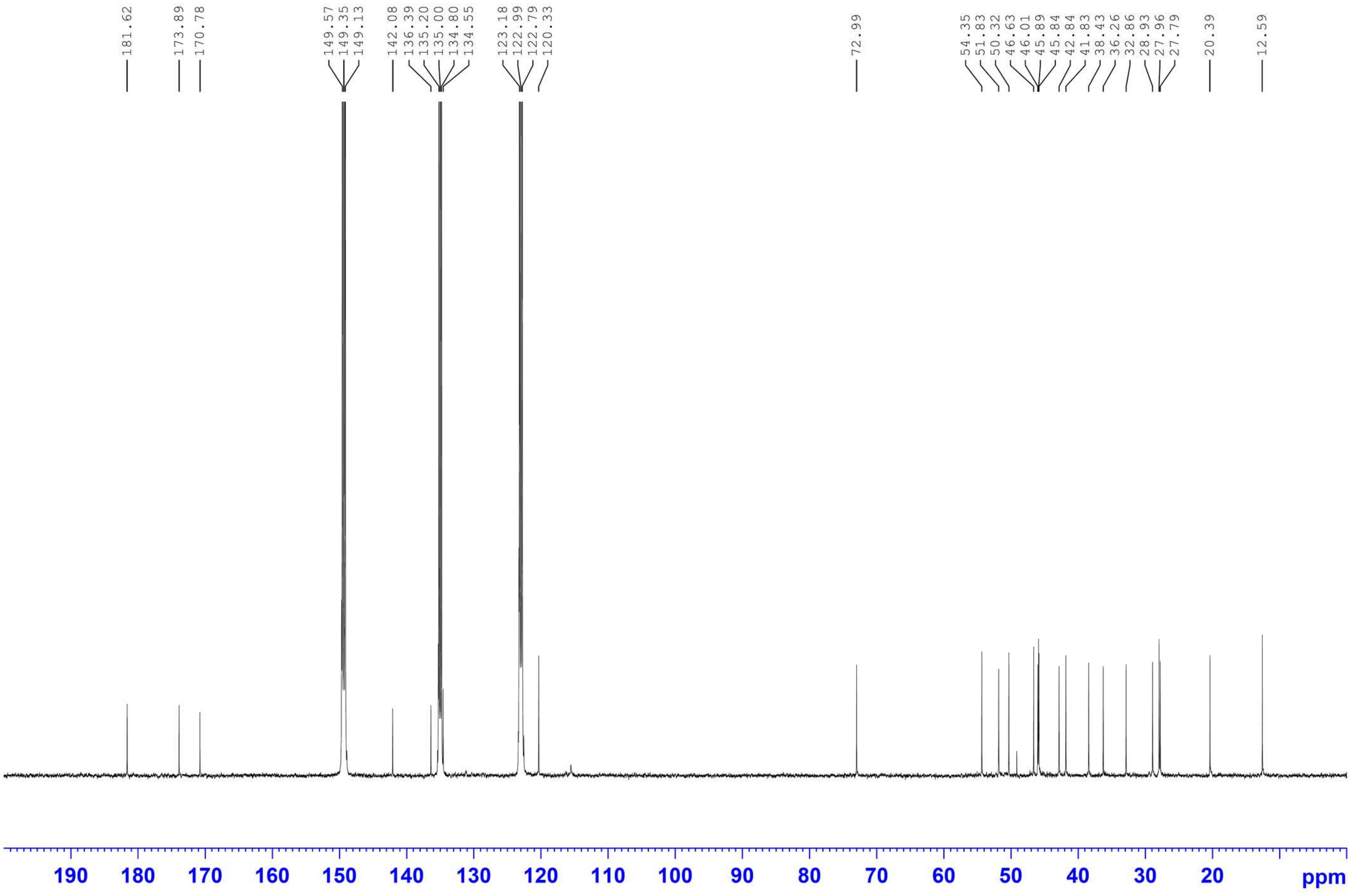

Figure S2-2. ^{13}C NMR spectrum (125 MHz) of compound **2** in pyridine-*d*5.

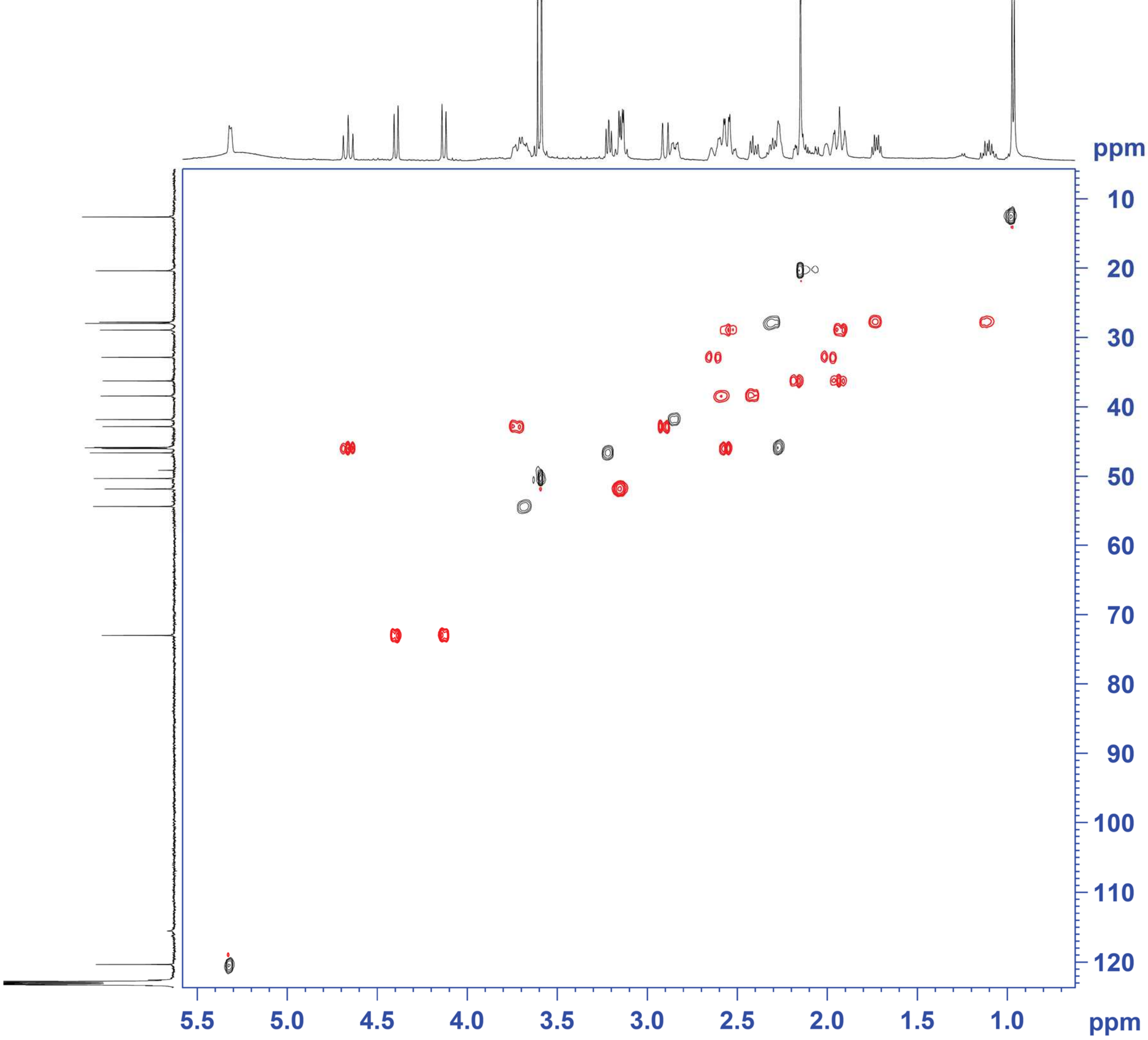

Figure S2-3. HSQC spectrum of compound **2** in pyridine-*d5*.

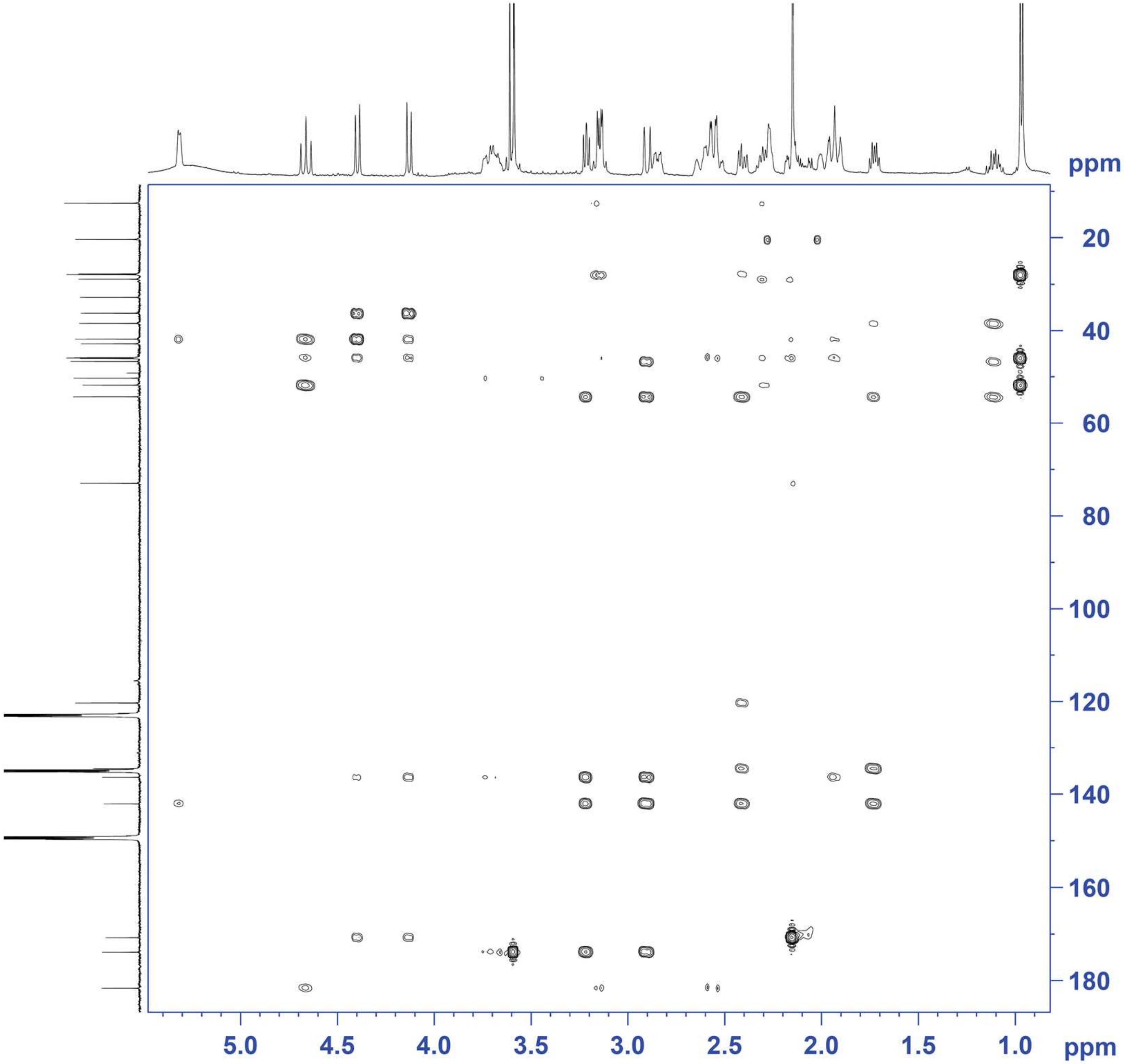

Figure S2-4. HMBC spectrum of compound **2** in pyridine-*d*5.

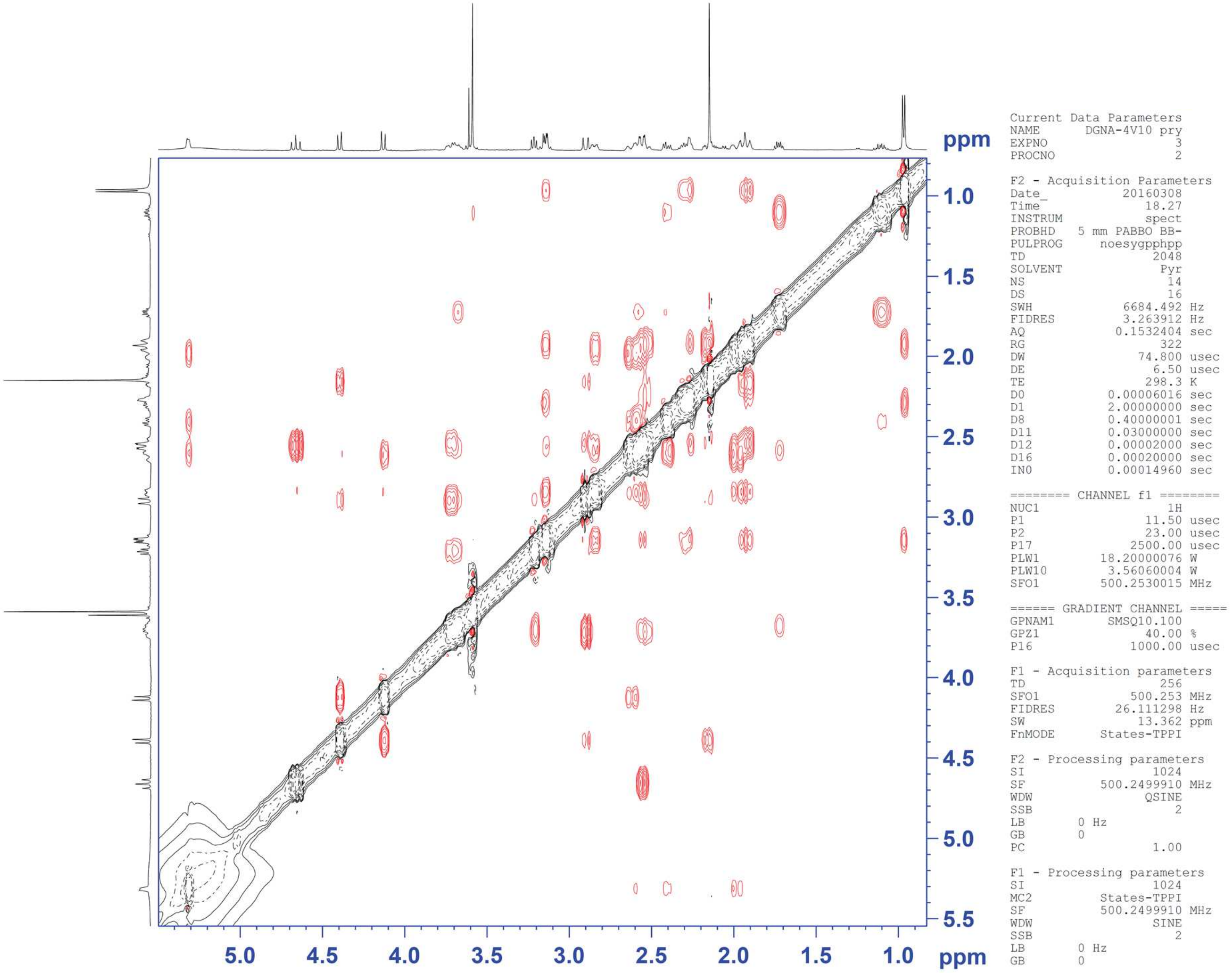

Figure S2-5. NOESY spectrum of compound **2** in pyridine-*d*5.

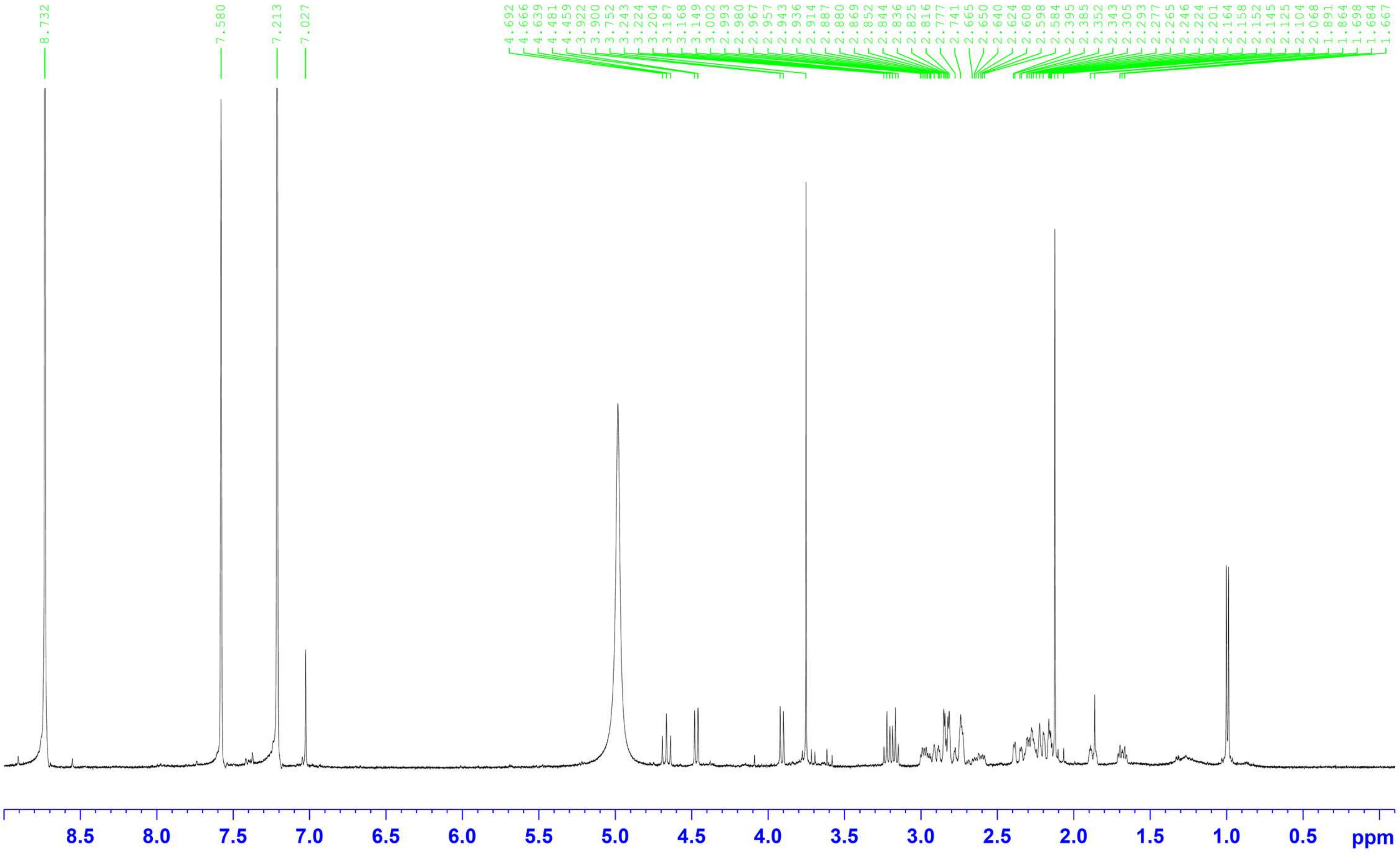

Figure S3-1. ^{1}H NMR spectrum (500 MHz) of compound **3** in pyridine-*d*5.

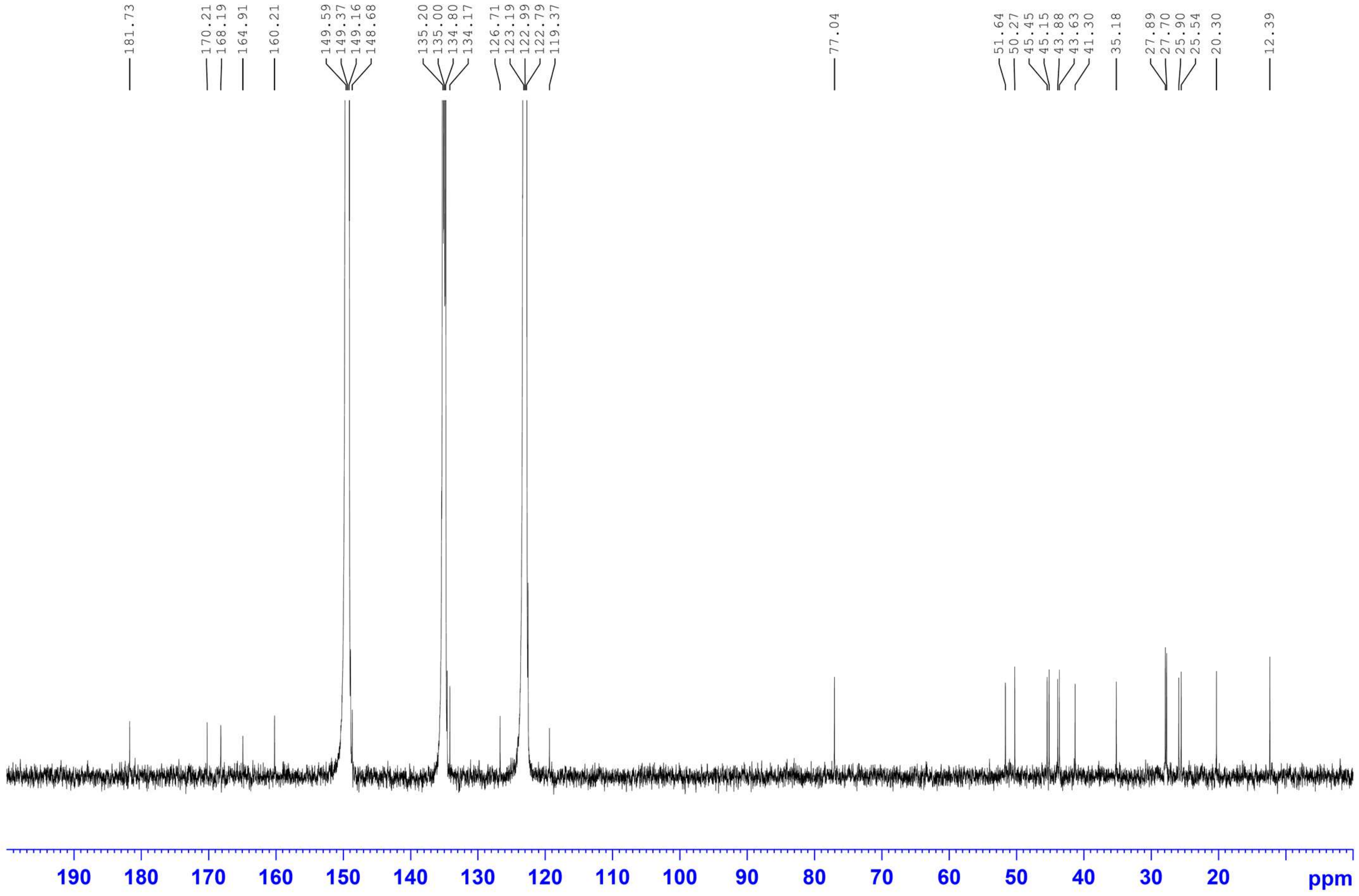

Figure S3-2. ^{13}C NMR spectrum (125 MHz) of compound **3** in pyridine-*d*5.

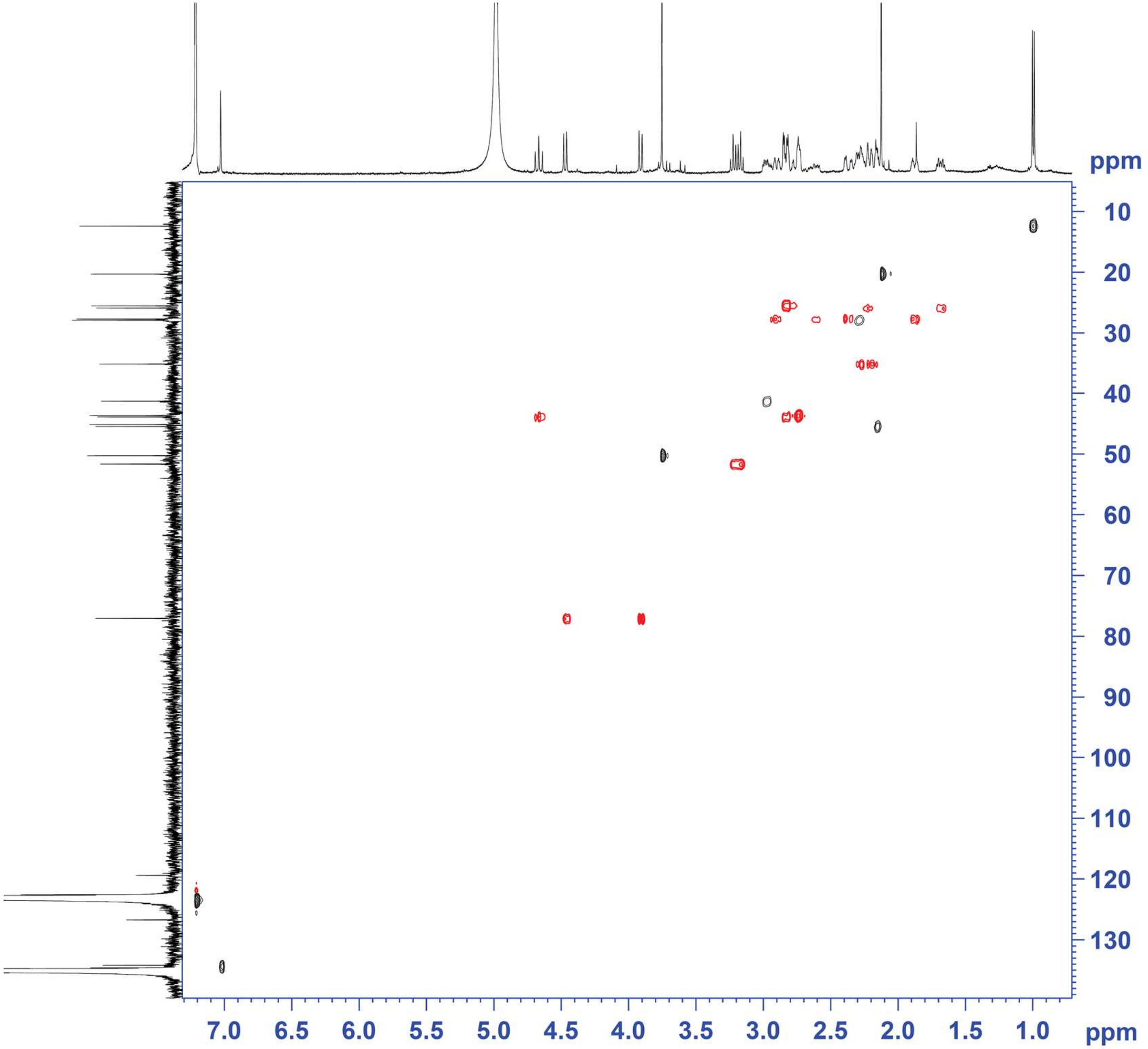

Figure S3-3. HSQC spectrum of compound **3** in pyridine-*d*5.

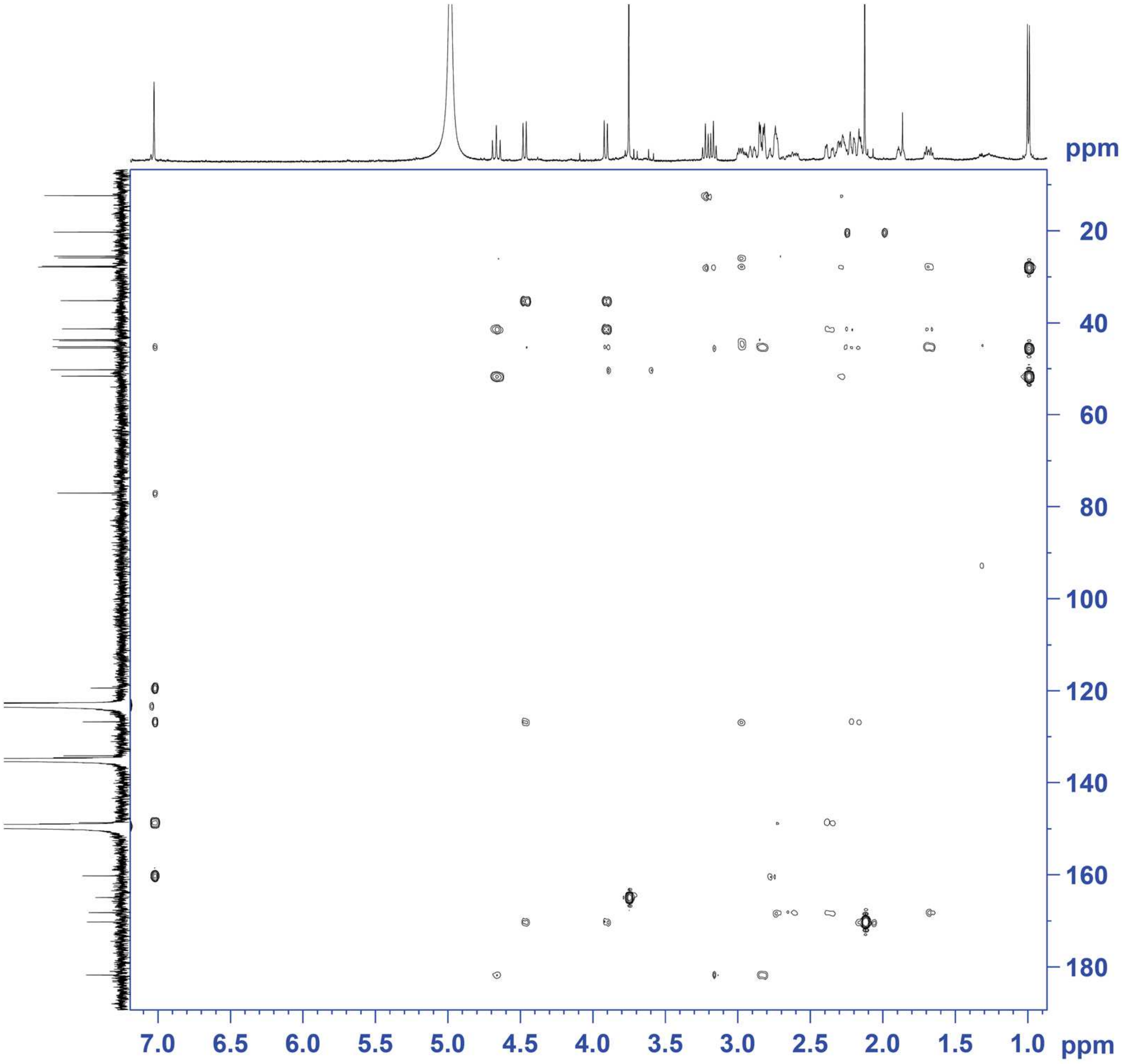

Figure S3-4. HMBC spectrum of compound **3** in pyridine-*d*5.

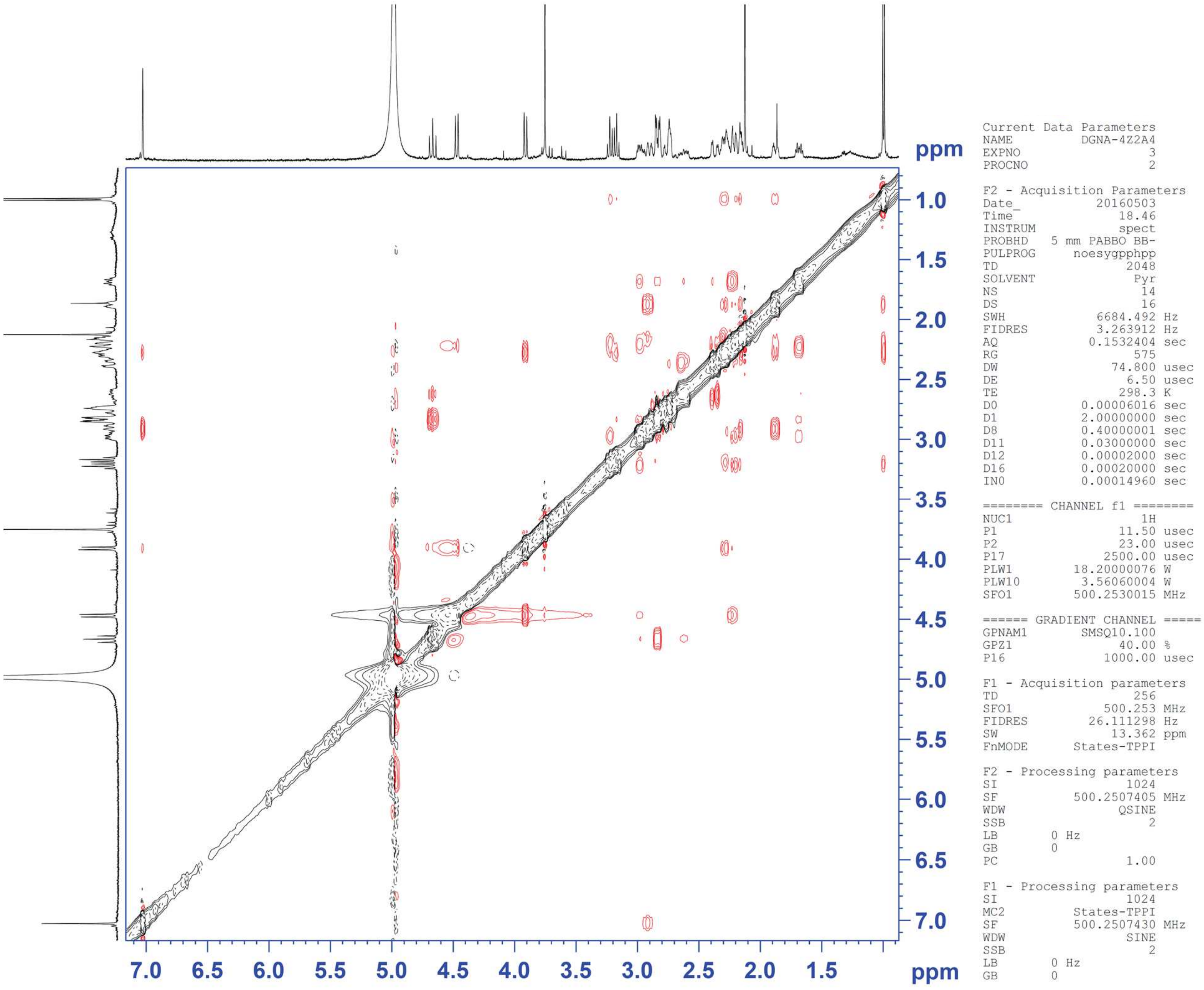

Figure S3-5. NOESY spectrum of compound **3** in pyridine-*d5*.

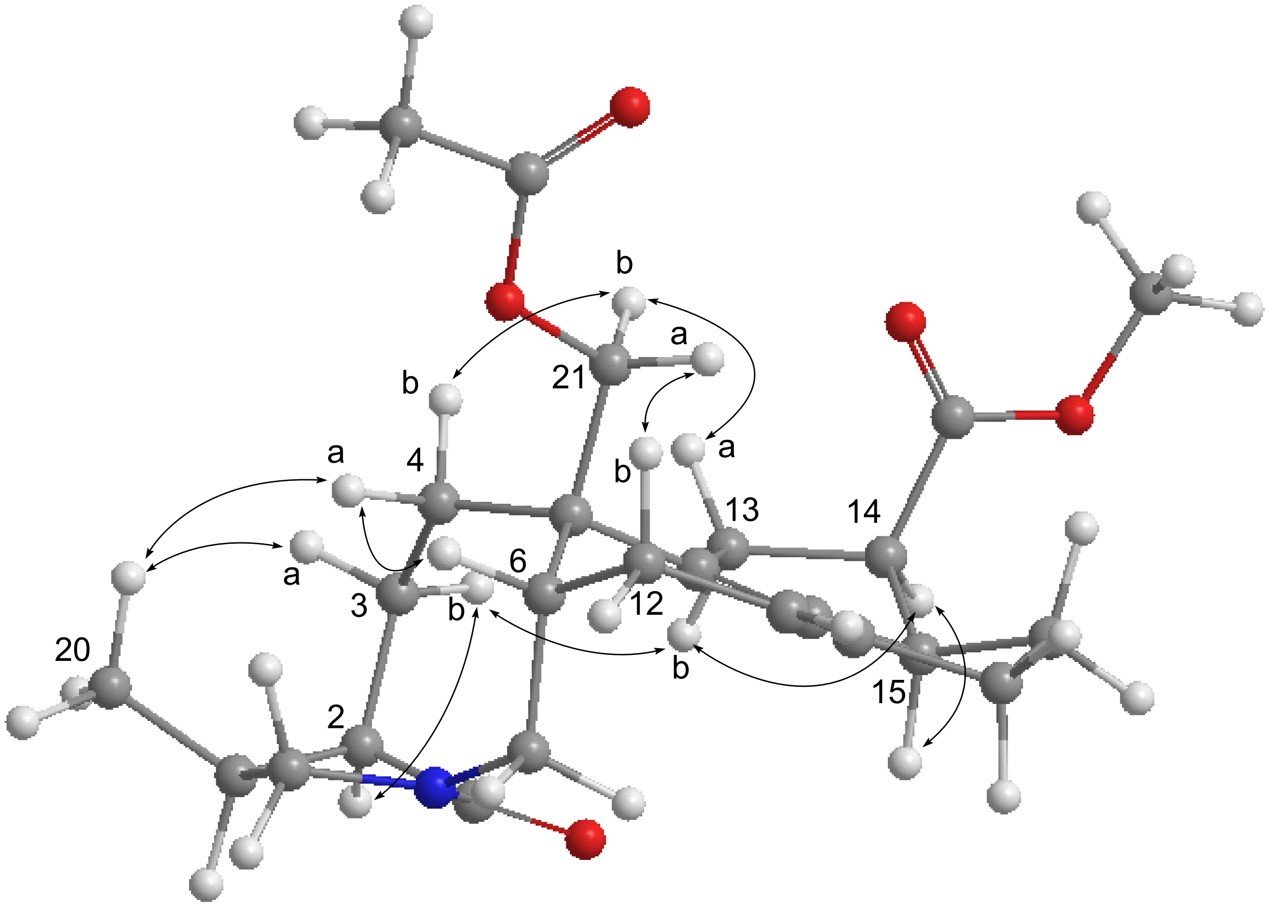

2

Figure S4. Selected NOESY correlations of **2**.

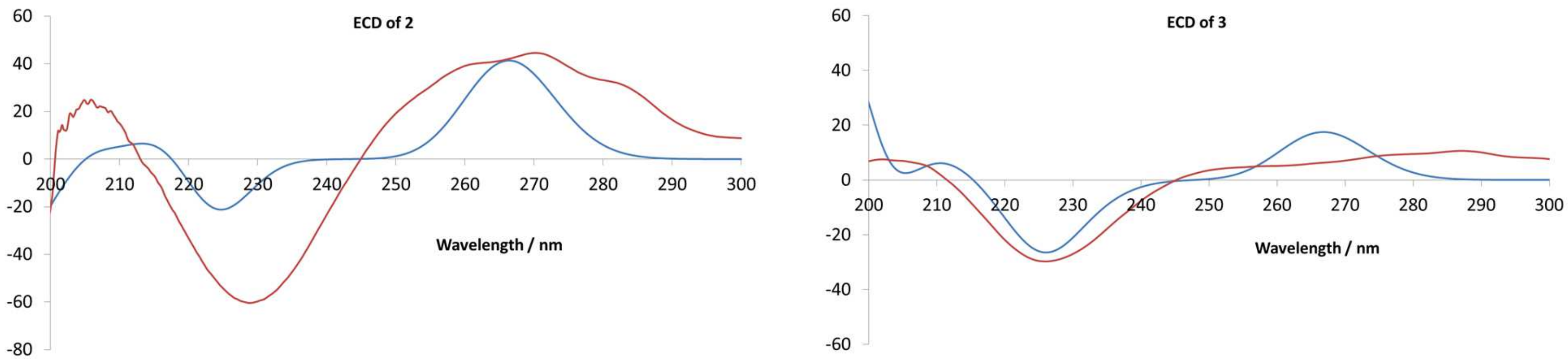

Figure S5. Experimental (red) and calculated (blue) ECD spectra of **2** (left) and **3** (right).

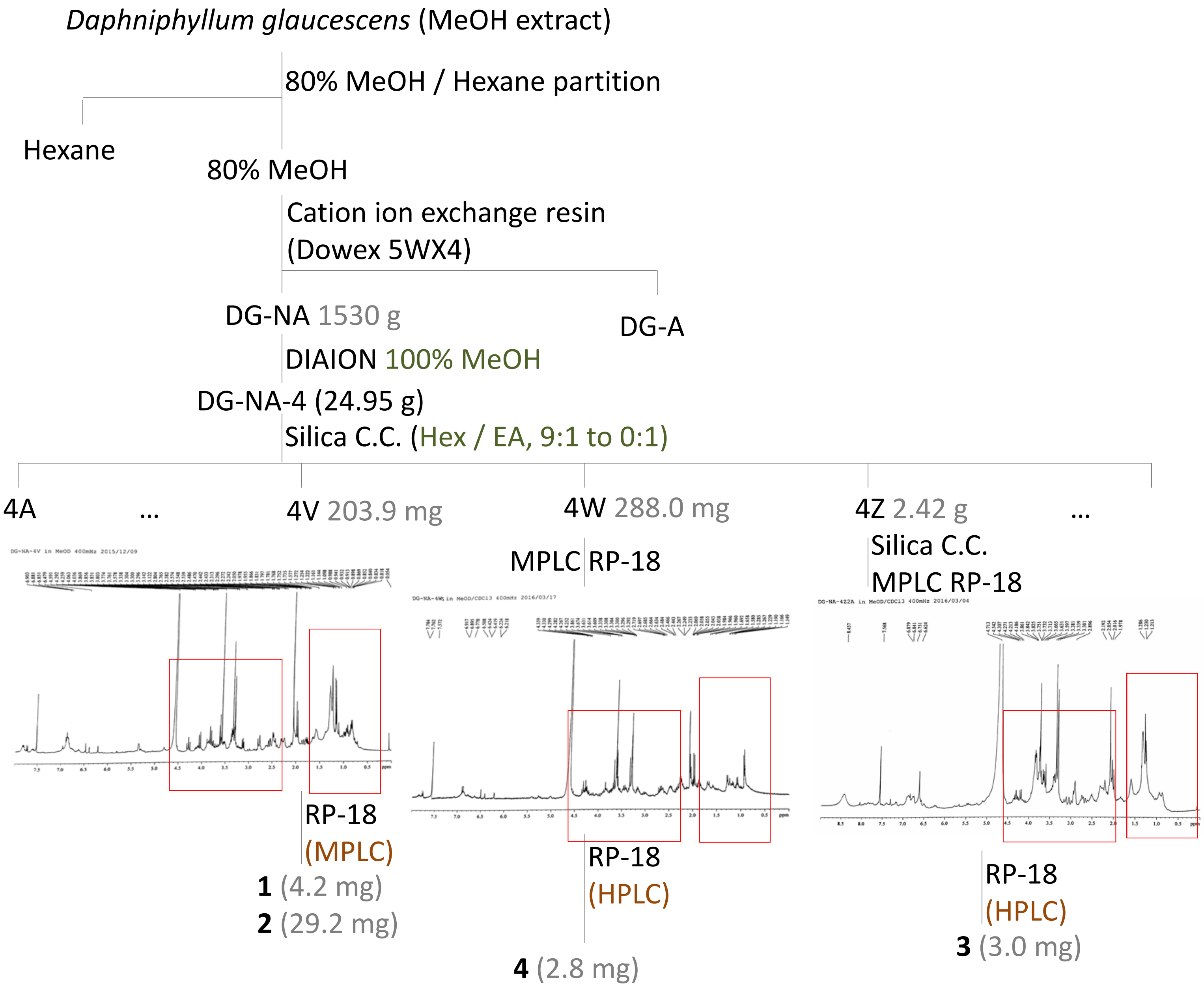

Figure S6. Isolation process using ion exchange resin and NMR fingerprint method.
